# Quercetin Feeding in Newborn Dairy Calves Cannot Compensate Colostrum Deprivation: Study on Metabolic, Antioxidative and Inflammatory Traits

**DOI:** 10.1371/journal.pone.0146932

**Published:** 2016-01-11

**Authors:** Jeannine Gruse, Ellen Kanitz, Joachim M. Weitzel, Armin Tuchscherer, Tadeusz Stefaniak, Paulina Jawor, Siegfried Wolffram, Harald M. Hammon

**Affiliations:** 1 Institute of Nutritional Physiology “Oskar Kellner”, Leibniz Institute for Farm Animal Biology (FBN), Dummerstorf, Germany; 2 Institute of Behavioural Physiology, Leibniz Institute for Farm Animal Biology (FBN), Dummerstorf, Germany; 3 Institute of Reproductive Biology, Leibniz Institute for Farm Animal Biology (FBN), Dummerstorf, Germany; 4 Institute of Genetics and Biometry, Leibniz Institute for Farm Animal Biology (FBN), Dummerstorf, Germany; 5 Department of Immunology, Pathophysiology and Veterinary Preventive Medicine, Faculty of Veterinary Medicine, Wroclaw University of Environmental and Life Sciences, Wroclaw, Poland; 6 Institute of Animal Nutrition and Physiology, Faculty of Agricultural and Nutritional Sciences, Christian-Albrechts-University of Kiel, Kiel, Germany; INIA, SPAIN

## Abstract

Immaturity of the neonatal immune system is causative for high morbidity in calves and colostrum intake is crucial for acquiring passive immunity. Pathogenesis is promoted by reactive oxygen species accumulating at birth if counter-regulation is inadequate. The flavonol quercetin exerts antioxidative and anti-inflammatory effects that may enhance neonatal health. The aim of this work was to study effects of quercetin feeding on metabolic, antioxidative and inflammatory parameters in neonatal calves to investigate whether quercetin could compensate for insufficient colostrum supply. Twenty-eight newborn calves were assigned to two dietary groups fed colostrum or milk-based formula on day 1 and 2 and milk replacer thereafter. From day 2 onwards, 7 calves per diet group were additionally fed quercetin aglycone (50 mg/(kg body weight × day)). Blood samples were taken repeatedly to measure plasma concentrations of flavonols, glucose, lactate, total protein, albumin, urea, non-esterified fatty acids, triglycerides, cholesterol, insulin, glucagon, cortisol, immunoglobulins, fibrinogen, haptoglobin and serum amyloid A. Trolox equivalent antioxidative capacity, ferric reducing ability of plasma, thiobarbituric acid reactive species and F2-isoprostanes were analyzed to evaluate plasma antioxidative status. Expression of tumor necrosis factor, interleukin-1α, interleukin-1β, serum amyloid A, haptoglobin, fibrinogen, C-reactive protein, catalase, glutathione peroxidase and superoxide dismutase mRNA were measured in liver tissue on day 8. Plasma flavonol concentrations were detectable only after quercetin-feeding without differences between colostrum and formula feeding. Plasma glucose, lactate, total protein, immunoglobulins, triglycerides, cholesterol, trolox equivalent antioxidative capacity and thiobarbituric acid reactive species were higher after colostrum feeding. Body temperature, fecal fluidity and plasma concentrations of cortisol and haptoglobin were higher in formula- than in colostrum-fed groups. Hepatic mRNA expression of tumor necrosis factor was higher after quercetin feeding and expression of C-reactive protein was higher after formula feeding. Data confirm that colostrum improves neonatal health and indicate that quercetin feeding cannot compensate for insufficient colostrum supply.

## Introduction

Calfhood diseases play a key role in the economy of dairy farms because they increase operating costs and reduce long-term productivity of the animal. Incidence of disease is associated with increased mortality rates [1], and enteritis is the most common diagnosis in young calves [2], which, according to Svensson, Linder and Olsson [3], contributes to 23% of calf losses during the first 14 days of life. Neonatal calves are prone to sickness because their immune system is immature. Furthermore, the process of birth itself causes an elevated stress level for the newborn and exposure to an oxygen-rich environment leads to an increased generation of reactive oxygen species [4, 5]. Reactive oxygen species induce peroxidation of lipids and other macromolecules, leading to alteration of cellular components, interaction with signaling cascades and modification of physiological cell functions [6]. If not properly counterbalanced by antioxidative defenses, excessive production of reactive oxygen species results in oxidative stress, which is a cofactor of disease in humans and farm animals [5, 7, 8]. Adequate colostrum supply is vital to calves because colostrum ensures ingestion of nutrients and contains immunoglobulins (Ig), peptides, antioxidants and other bioactive factors supporting maturation, antioxidative and immune defense as well as local intestinal immunity [9].

The ban on antibiotic performance promoters by the European Union in 2006 increased efforts to establish natural alternatives to enhance health and productivity in breeding. Special focus has been directed to phytochemicals because their use can be manifold according to the respective compound [10]. Flavonoids are secondary plant metabolites that are widely distributed in the plant kingdom and are able to modulate inflammation and immune function and exert antioxidative activity [11–13].

Quercetin, which belongs to the subclass of flavonols, is ubiquitous in most plants and is of interest for scientists for its beneficial use in humans and farm animals. Its antioxidative capacity can ameliorate the acquisition of passive immunity in neonates, based on the finding that feeding antioxidant-enriched colostrum enhanced IgG absorption and antioxidative status in newborn calves and piglets [14, 15]. Similarly, Retskii et al. [16] showed that correcting the antioxidative balance in newborn calves prior to first colostrum ingestion increases the acquisition of colostral immunity and reduces the incidence of enteric colibacillosis. Another beneficial effect of quercetin is its local action in the gastrointestinal tract. *In vitro* studies of intestinal epithelium demonstrated that quercetin down-regulates the expression of genes related to inflammation in inflamed epithelium [17], and Lozoya et al. [18] showed in a clinical study that oral quercetin administration reduced abdominal pain in acute diarrheic disease in humans. In guinea pigs, mice and rats, the inhibitory action of quercetin on prostaglandin E_2_-induced ileal contractions and on castor-oil-induced diarrhea has been demonstrated [19, 20]. Furthermore, quercetin acts as a prebiotic, thus inhibiting adhesion of enteropathogens to Caco-2 cells without affecting the viability of probiotics [21], and improves performance in hens by modulating cecal microflora populations [22].

Although a multitude of research on quercetin has been performed *in vitro* or in animal models for medical conditions, studies of the effects in neonatal farm animals are scarce. The aim of the present work was to investigate the potential health-promoting effects of feeding quercetin to newborn calves during the first week of life and to evaluate whether the health-promoting effects of quercetin compensate for initial colostrum deprivation in calves. We hypothesized that quercetin improves antioxidative balance and immune function and that local antibacterial and anti-inflammatory effects reduce the incidence of diarrhea and gastrointestinal dysfunctions.

## Materials and Methods

### Animals, Husbandry and Feeding

Experimental procedures were conducted in compliance with the German Animal Protection regulations with approval of the authorities of the state Mecklenburg-Western Pomerania, Germany (Landesamt für Landwirtschaft, Lebensmittelsicherheit und Fischerei Mecklenburg-Vorpommern; LALLF M-V/TSD/7221.3–1.1-044/12). Liver biopsies were performed under local lidocaine anesthesia and all calves received metamizole post-operatively for pain relief.

Twenty-eight male Holstein Friesian calves were separated from their dams immediately after birth and were housed in single boxes during their first 8 days of life. Before the trial started, separate colostrum pools were prepared from the first and third milkings after parturition. According to the colostrums’ macronutrient compositions, milk-based formulas with comparable amounts of macronutrients [23] but without bioactive factors were provided (Bergin MAT-Formula; Bergophor Futtermittelfabrik, Kulmbach, Germany). Calves were randomly assigned to two dietary groups and were bucket-fed twice daily, receiving either colostrum (Col, n = 14) or corresponding formula (For, n = 14) on days 1 and 2 of life (10% and 12% of body weight/day, respectively). If appetite was reduced, calves were tube fed to ensure complete ingestion of colostrum or formula. From day 3 until day 8, all calves were fed commercial milk replacer (12% of body weight/day; 150 g/L; SALVAlac MIRApro 45; Salvana Tiernahrung, Klein-Offenseth Sparrieshoop, Germany).

On day 2, the dietary groups were subdivided into control (ColQ- and ForQ-; n = 7 per group) and treatment groups (ColQ+ and ForQ+; n = 7 per group), the latter receiving quercetin aglycone twice daily with feeding (50 mg/(kg body weight × day); quercetin aglycone dihydrate ≥98%, Carl Roth, Karlsruhe, Germany). The control groups received no quercetin aglycone.

#### Treatment

Navels were disinfected with 10% povidone iodine solution (Vet-Sept; aniMedica, Senden-Bösensell, Germany). Neonatal calves received an oral dose of 1 g iron dextran with their first meal on day 1 (Ursoferran; Serumwerk Bernburg, Bernburg, Germany). To support immunological defense during the first 5 days of life, all calves received chicken egg-derived immunoglobulins with the morning feeding (0.25 g/kg body weight; Globigen Life Start 25%, EW Nutrition, Visbek, Germany) [24]. To prevent cryptosporidiosis, calves were treated with halofuginone (0.1 mg/kg body weight per *os*; Halocur, Intervet, Igoville, France) after the evening feeding from day 1 to day 7.

Colostrum-deprived calves (ForQ+, ForQ-) additionally received B-vitamins (100 mg nicotinamide/calf, 40 mg thiamin chlor*i*de hydrochloride/calf, s.c.; Vitamin-B-Komplex, Serumwerk Bernburg, Germany) and bovine colostral immunoglobulins on days 1 (s.c.), 3 and 5 (per *os)* (2 g gammaglobulins/calf with antibodies against *Escherichia coli*, rotavirus, coronavirus; Aniserin orinject; aniMedica, Seden-Börsensell, Germany). Furthermore, formula-fed calves were treated metaphylactically with colistin sulfate from day 2 to day 8 (3 mg/kg body weight, i.m.; Belacol; BelaPharm, Vechta, Germany).

All calves were weighed immediately after birth and before evening meals on days 2 and 6. Every morning, health status was examined and appetite, general condition, heart rate, respiratory rate, rectal temperature and gut motility were assessed. Fecal fluidity was scored according to Larson et al. [25]. Calves with reduced vitality after the first 2 days of life were allowed one recovery day before further sample taking. In these cases, the times referred to as days 3, 4, 7 and 8 in the results section are days 4, 5, 8 and 9 after birth, respectively. Due to gastrointestinal imbalances, two calves (one calf of group ColQ+ and one calf of group ForQ+) had to be excluded from the study.

### Blood Analyses

#### Sample Taking

Basal blood samples were taken before the morning feeding on days 1, 2, 4 and 7 from the jugular vein using evacuated tubes containing either potassium-EDTA (1.2–2 mg/mL EDTA) for analyses of plasma metabolites, insulin, glucagon, immunoglobulins and acute-phase proteins or Li-heparin (12–30 IU heparin) for the determination of the cortisol and flavonol concentrations and the antioxidative status in the plasma. For flavonol analysis, additional blood samples were taken before the morning feeding on days 3 and 8. After centrifugation (1,500 × *g*, 4°C, 20 min), plasma aliquots were stored at -20°C until analyses (-80°C for analyses of flavonol concentrations and antioxidative status, respectively).

#### Plasma Flavonols, Metabolites and Hormones

Plasma concentrations of flavonols (quercetin, isorhamnetin, tamarixetin and kaempferol) were measured via HPLC as previously described [26] with a detection limit of 2 nmol/L and a recovery rate of 92 ± 2%. The intra- and inter-assay coefficients were 0.5 and 7.2%, respectively.

Plasma metabolites were analyzed using an automatic spectrophotometer (ABX Pentra 400; Horiba ABX, Montpellier, France) and respective kits: glucose (#A11A01667), lactate (#A11A01721), albumin (#A11A01664) and triacylglycerides (#A11A01640) from HORIBA ABX, Montpellier, France; total protein (#553–412) and cholesterol (#553–127) from mti-diagnostics, Idstein, Germany; urea (#LT-UR 0010) from Labor+Technik, E. Lehmann, Berlin, Germany; and non-esterified fatty acids (#434-91795, #436-91995) from WAKO Chemicals, Neuss, Germany.

Plasma concentrations of insulin (#RIA-1257) and glucagon (#RIA-1258) were determined by RIA using kits from DRG Instruments, Marburg, Germany, which were adapted to bovines [27]. Intra- and inter-assay coefficients of variation were 3.7% and 5.5% for insulin and 3.4% and 22.5% for glucagon, respectively. Plasma cortisol concentrations were analyzed in duplicate after extraction with diethylether using a commercially available ELISA kit (#EIA1887; DRG Instruments GmbH, Marburg, Germany) according to the instructions of the manufacturer. Cross reactivities of the antibody to corticosterone and progesterone were 45% and 9%, respectively, and <2% to any further competing plasma steroids. The assay was validated for use with bovine plasma. The test sensitivity was 3.4 μg/L, and intra- and inter-assay coefficients of variation were 5.3% and 12.1%.

#### Immunoglobulins (Ig) and Acute-Phase Proteins

The concentrations of IgG1, IgG2 and IgM, as well as acute-phase proteins (haptoglobin, serum amyloid A and fibrinogen), were measured in the EDTA plasma samples taken on days 1, 2, 4 and 7. IgG1 was analyzed by radial immunodiffusion [28] (modified by Gasowska and Stefaniak [29]) using bovine reference serum (RS10-103; Bethyl Laboratories Inc., Montgomery, USA) as standard. IgG2 (#E10-117) and IgM (#E10-101) were determined by ELISA using kits from Bethyl Laboratories Inc., Montgomery, USA. Intra-assay coefficients of variation were 10.9% and 4.0% for IgG2 and IgM, respectively. The detection limit of IgG2 was 7.8 μg/L and that of IgM was 15.6 μg/L. For detection of serum amyloid A (SAA; #TP-802), we used a multispecies ELISA kit from Tridelta Development, Maynooth, Ireland. The detection limit of SAA was 9.4 mg/L. The intra-assay coefficient of variation was 12.0%. The haptoglobin concentration was analyzed using the guaiacol method developed by Jones and Mould [30] with human haptoglobin Hp 2–2 (Sigma #H9762) as a standard. The detection limit of haptoglobin was 0.01 g/L. Plasma fibrinogen was determined by rapid heat precipitation according to Millar, Simpson and Stalker [31].

#### Antioxidative Status

Li-heparinized plasma samples taken on days 1, 4 and 7 were used to analyze the Trolox-Equivalent Antioxidative Capacity (TEAC, as trolox equivalents (TE) in mmol/L) and Ferric Reducing Ability of Plasma (FRAP, as Ascorbic Acid Equivalents, (ASCE) in μmol/L) as parameters of antioxidative capacity as well as Thio-Barbituric Acid Reactive Species (TBARS, as Malondialdehyde Equivalents (MDAE) in μmol/L) and 8-iso-PGF_2α_ (F2-isoprostanes, in ng/L) as markers for oxidative stress.

TEAC was analyzed as described by Miller et al. [32] and modified by Re et al. [33]. FRAP and TBARS were determined according to Luehring et al. [34]. For determination of F2-isoprostanes, a commercial ELISA kit (#ADI-900-091; Enzo Life Sciences, Lause, Switzerland) was used. Cross-reactivities of the assay to PGF_1α_ and PGF_2α_ were 4.6% and 1.85%, respectively, and <1% to any further eicosanoids.

### Liver Tissue Analyses

On day 8, a liver biopsy was conducted 2 h after the morning meal using a custom-made biopsy trocar [23]. Biopsy tissue was immediately frozen in liquid nitrogen and stored at -80°C until further analysis. Hippocalcin-like 1 (*HPCAL1*; NM_001098964), low-density lipoprotein 10 (*LRP10*; BC149232) and RNA polymerase II (*POLR2A*; NM_001206313.1) were used as reference genes (given accession numbers related to NIH GenBank). Primer sequences, accession numbers and PCR conditions for target genes related to antioxidative status (catalase (*CAT*); glutathione peroxidase (*GPX1*); superoxide dismutase (*SOD*)) and inflammation (tumor necrosis factor (*TNF*); interleukin-1α and -1β (*IL1A*, *IL1B*); haptoglobin (*HP*); fibrinogen (*FGA*); serum amyloid A2 (*SAA2*); and C-reactive protein (*CRP*)) are listed in Table 1. As recently described [23], primer products were verified by sequencing using the BigDye Terminator v1.1 Cycle Sequencing kit and an ABI 3130 Genetic Analyzer (Life Technologies, Carlsbad, USA). Real-time PCR was performed using a LightCycler (Roche Molecular Biochemicals, Mannheim, Germany); SYBR Green I was used as the fluorescent dye. Melting curve analysis and agarose gel electrophoresis were used to confirm the specificity of the PCR products. Quantification cycle values and amplification efficiencies obtained using LinRegPCR version 2013.0 [35] were imported into qBASE+ version 2.6.1 (Biogazelle, Gent, Belgium) for all subsequent calculations and quality controls. The geometric mean of the reference gene abundances was used for normalization. Data are presented as the ratio of the copy numbers of genes of interest and the geometric mean of the reference genes’ abundances.

**Table 1:** Characteristics of Primers and Real-Time RT-PCR Conditions^1^.

Gene	Forward (5’→ 3’)	Reverse (5‘→ 3’)	NIH GenBank accession number	Amplicon size (bp)	Mean Cq^2^	Efficiency
**Proinflammatory Cytokines**						
*TNF*	AGAGGGAAGAGCAGTCCCCAG	TTCACACCGTTGGCCATGAG	NM_173966.3	181	27.30	1.90
*IL1A*	TGAACGACGCCCTCAATCAA	GGTGTCTCAGGCATCTCCTTT	NM_174092.1	226	27.98	1.90
*IL1B*	AACGTCCTCCGACGAGTTTC	GCTCATGCAGAACACCACTTC	NM_174093.1	163	25.70	1.89
**Acute-Phase Proteins**						
*HP*	GGCCCCCGAGATTGCTAATA	CTCTGGGCAGCTGTCATCTT	NM_001040470.2	172	15.53	1.88
*SAA2*	CCACTGGGGATCAGCACAAT	CCTCTTTGGGCAGCGTCATA	NM_001075260.2	212	16.25	1.82
*FGA*	CGCGATTGAAAGCAAGCACT	GAAGTGTGGATACCTCTGGCA	NM_001033626.1	129	14.71	1.87
*CRP*	CAGGCCAGACAGACTTGCATA	TGCTGCTTGGTGGCATAA	NM_001144097.1	181	18.52	1.90
**Antioxidative Enzymes**						
*CAT*	TCACTCAGGTGCGGACTTTC	GGATGCGGGAGCCATATTCA	NM_001035386.2	162	18.40	1.87
*GPX1*	CTTCCCCTGCAACCAGTT	GGCAATTCAGGATCTCCTCGTT	X13684.1	62	20.97	1.87
*SOD*	AAGGCCGTGTGCGTGCTGAA	CAGGTCTCCAACATGCCTCT	M81129.1	246	20.44	1.87

^1^ Initial denaturation = 10 min at 95°C; denaturation = 15 s at 95°C; annealing = 10 s at 60°C; extension = 30 s at 72°C.

^2^ Quantification cycle.

### Statistical Analyses

Statistical analyses were conducted using SAS software, Version 9.3 for Windows, SAS Institute Inc., Cary, USA. Descriptive statistics and tests for normality were calculated using the UNIVARIATE procedure of Base SAS software. Body weight, average daily weight gain and data for hepatic gene expression were analyzed by ANOVA with the MIXED procedure of SAS/STAT taking a model with the fixed factors diet (levels: Col vs. For), quercetin (levels: Q+ vs. Q-) and the interaction diet×quercetin. Feed intake, body temperature, heart and respiratory rate and plasma concentrations of metabolites, hormones, flavonols and markers of antioxidative status were analyzed by repeated measurement ANOVA using the MIXED procedure of SAS/STAT software and a model with the fixed factors diet, quercetin and day of life (repeated variable) and all interactions between the fixed factors. Repeated measures on the same calf were taken into account using the REPEATED statement of the MIXED procedure and a type for the block diagonal residual covariance matrix chosen in dependence on the levels of day of life. For concentrations of plasma metabolites, hormones and data on antioxidative status in plasma, an unstructured type was used. For the plasma concentrations of flavonols, acute-phase proteins and Ig, a compound symmetry type was used. For data on heart and respiratory rate, as well as body temperature and feed intake, an autoregressive (1) type was applied. Least-squares means (LSM) and their standard errors (SE) were computed for each fixed effect in the models and all pairwise differences of LSM were tested by the Tukey-Kramer procedure. The SLICE statement of the MIXED procedure was used to conduct partitioned analyses of the LSM for interactions. Fecal score was analyzed by a generalized linear mixed model using the GLIMMIX procedure of SAS/STAT and a Poisson model with the fixed factors diet, quercetin and day of life (repeated variable) and all interactions between these fixed factors. Repeated measurements on the same animal were taken into account by the RESIDUAL option of the RANDOM STATEMENT of the GLIMMIX procedure using a compound symmetry structure for the block diagonal residual covariance matrix. Sick frequencies of calves with respect to diet and quercetin were analyzed with the FREQ procedure of SAS/STAT software using two-way tables of diet by sick and quercetin by sick and the exact Pearson chi-square test.

Effects and differences were considered significant at *P* < 0.05.

## Results

### Feeding, Growth Performance and Health Status

**Fig 1:**
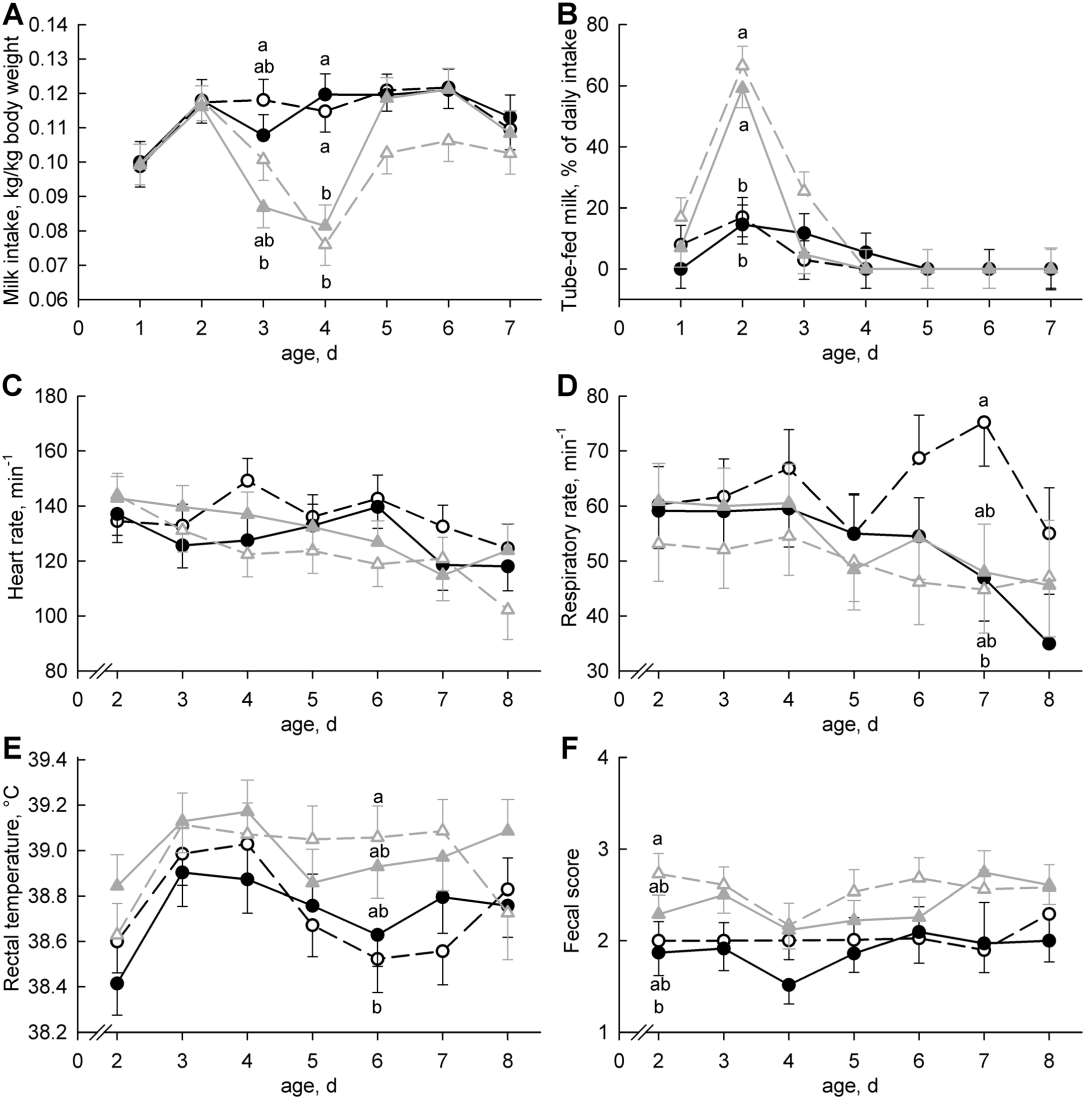
Feed Intake and Health Parameters. Neonatal calves were fed either colostrum (black circles) or formula (gray triangles) on days 1 and 2 and were supplemented with (filled symbols, solid lines) or without (open symbols, dashed lines) quercetin aglycone from day 2 to day 8 (50 mg/(kg body weight × day)). (**A**) Milk intake, (**B**) percent milk intake by tube feeding, (**C**) heart rate, (**D**) respiratory rate, (**E**) body temperature and (**F**) fecal score (according to Larson et al. [25]; 1 = normal, 2 = soft, 3 = runny, 4 = watery) were observed daily. Data are presented as the least squares means ± standard errors. Least squares means with different lowercase letters (a, b) differ among groups within the same day (*P* < 0.05).

Mean body weight at birth was 45.5 ± 1.9 kg and increased with age (*P* < 0.01) by 368 ± 140 g/d, without group differences, respectively. All calves received their first meal 2.2 ± 0.1 h after birth. Milk intake related to body weight did not differ among groups on days 1 and 2 but was lower on days 3 and 4 in the colostrum-deprived calves (*P* < 0.01; Fig 1A). On day 2, appetite was reduced in the formula-fed calves and the amount of tube-fed milk was higher than in colostrum-fed calves (*P* < 0.01; Fig 1B). Heart rate decreased with age (*P* < 0.01) and respiratory rate tended to decrease (*P* = 0.08; Fig 1C and 1D). Rectal temperature was highest on days 3 and 4 and subsequently decreased (*P* < 0.01). Rectal temperature and fecal score were higher in formula-fed than in colostrum-fed calves (*P* < 0.01; Fig 1E and 1F). The number of calves with an allowed recovery day was similar among groups. With the exception of ColQ+, we treated one calf per group medically for navel infection. Four calves in group ForQ+ needed antispasmodic/analgesic treatment during a recovery day because of abdominal pain. Thus, Col-fed calves tended to be less susceptible to illness (*P* = 0.08), whereas quercetin treatment did not affect well-being (*P* = 0.38). Due to severe disease, we had to remove two calves from the study on day 4 (ForQ+) and day 7 (ColQ+).

### Flavonoid Content

**Fig 2:**
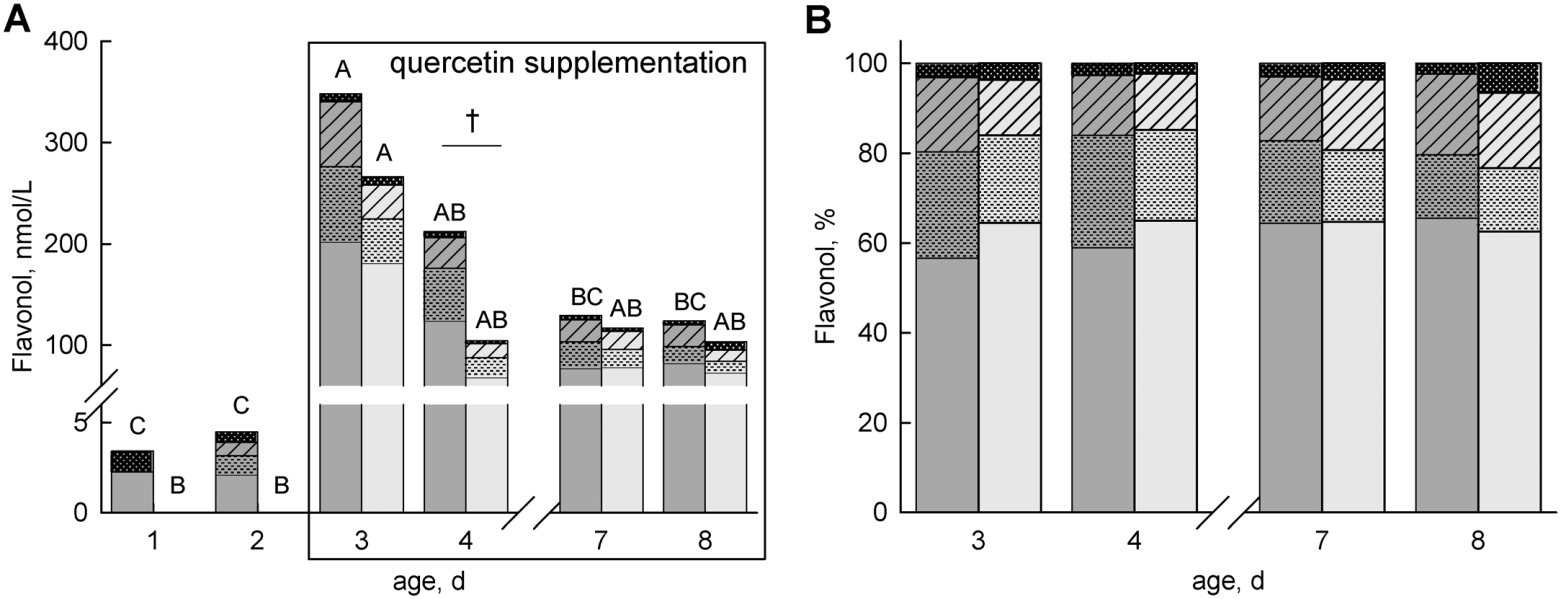
Flavonol concentrations in plasma. Stacked concentrations (**A**) and percentage composition (**B**) of flavonols in basal plasma samples of calves fed either colostrum (dark grey) or formula (light grey) on days 1 and 2 and supplemented with quercetin aglycone from day 2 to day 8 (50 mg/(kg body weight × day)). Flavonol metabolites: quercetin (without symbol), isorhamnetin (horizontal line), tamarixetin (crossline) and kaempferol (horizontal bold line). Data are presented as the least squares means. Different uppercase letters (A, B, C) symbolize differences in the total flavonol concentration within the same group on different days (*P* < 0.05). † tend to differ among groups on the same day (*P* < 0.10).

Preprandial concentrations of total flavonols in plasma on days 1 and 2 (before first quercetin supplementation) did not reveal significant differences among groups, but very low concentrations were detectable in five colostrum-fed calves. In the control groups ForQ- and ColQ-, the plasma concentrations did not change with age. In ColQ+ and ForQ+, the plasma flavonol concentrations changed with age (*P* < 0.01); they reached the maximum on day 3 and decreased subsequently but tended to differ among groups only on day 4 (*P* = 0.08; Fig 2A). Comparing the flavonol fractions in the plasma, the quercetin fraction (60% of total flavonoids) was highest in both dietary groups. The concentrations of isorhamnetin and tamarixetin on day 3 were higher in ColQ+ than in ForQ+ (Fig 2B).

### Plasma Metabolites and Hormones

**Fig 3:**
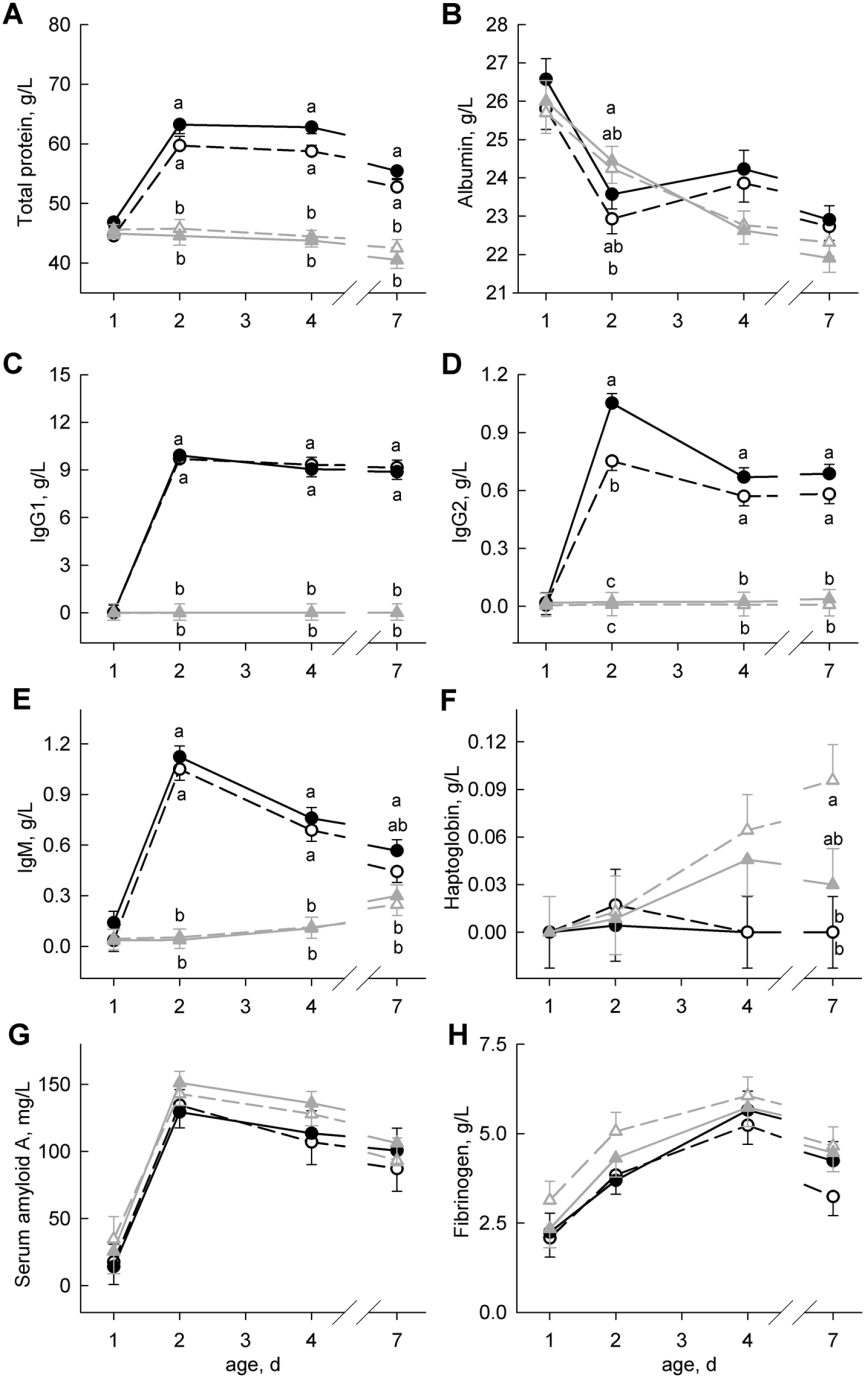
Total Protein, Albumin and Immune and Inflammatory Status in Blood Plasma. Basal concentrations of total protein (**A**), albumin (**B**), immunoglobulins G1, G2 and M (**C**, **D**, **E**) and acute-phase proteins haptoglobin, serum amyloid A and fibrinogen (**F**, **G**, **H**) in the plasma of calves fed either colostrum (black circles) or formula (gray triangles) on days 1 and 2 and supplemented with (filled symbols, solid lines) or without (open symbols, dashed lines) quercetin aglycone (50 mg/(kg body weight × day)) from day 2 to day 8. Data are presented as the least squares means ± standard errors. Least squares means with different lowercase letters (a, b, c) differ among groups within the same day (*P* < 0.05).

Plasma glucose concentrations were lowest on day 1, increased on day 2 in ColQ-, ForQ+ and ForQ- (*P* < 0.05) and decreased on day 4 only in ForQ+ and ForQ- (*P* < 0.05). The mean plasma concentration of glucose was higher in the colostrum-fed than in the formula-fed calves (*P* < 0.03; Table 2). The plasma lactate and non-esterified fatty acid concentrations were highest on day 1 and decreased with age (*P* < 0.01) without any group differences (Table 2). The concentrations of total protein in the plasma increased only in the colostrum-fed calves (*P* < 0.01) and were higher in the colostrum-fed than in the formula-fed calves on day 2, day 4 and day 7 (*P* < 0.01; Fig 3A). Plasma albumin decreased with age (*P* < 0.01) in all groups (Fig 3B). For both total protein and albumin, the diet×age interaction was significant (*P* < 0.01). The plasma urea concentrations increased on day 2 in the formula-fed and on day 7 in the colostrum-fed calves (*P* < 0.01; Table 2). The plasma triglyceride concentrations increased until day 4 only in the colostrum-fed calves (*P* < 0.05) and continuously decreased from day 2 to day 7 in the formula-fed calves (*P* < 0.01). The plasma cholesterol concentration increased with age (*P* < 0.01). The plasma triglyceride (day 7) and cholesterol concentrations (day 4 and day 7) were higher in the colostrum-fed than in the formula-fed calves (*P* < 0.01; Table 2).

**Table 2:** Metabolites and Hormones. Data are given as the least squares means ± standard errors. Least squares means within a row with different lowercase letters (a, b) differ (*P* < 0.05). Q+, quercetin-supplemented; Q-, control (no quercetin); n = 7 per group.

	Age	Group (Diet, Quercetin)				ANOVA *P*-values^1^			
		Colostrum		Formula					
Item	(d)	Q-	Q+	Q-	Q+	Diet	Quercetin	Age	Diet × Age
Glucose (mmol/L)	1	4.3 ± 0.6	4.7 ± 0.6	4.1 ± 0.6	4.1 ± 0.6	0.03	0.89	<0.01	0.06
	2	6.0 ± 0.4	5.5 ± 0.4	5.8 ± 0.4	5.8 ± 0.4				
	4	5.5 ± 0.2^a^	5.6 ± 0.2^a^	4.4 ± 0.2^b^	4.4 ± 0.2^b^				
	7	5.2 ± 0.2^a^	5.2 ± 0.2^a^	4.4 ± 0.2^b^	4.6 ± 0.2^ab^				
Lactate (mmol/L)	1	4.0 ± 0.7	2.8 ± 0.7	3.7 ± 0.7	3.1 ± 0.7	0.88	0.78	<0.01	0.32
	2	2.4 ± 0.3	3.0 ± 0.3	2.7 ± 0.3	2.9 ± 0.3				
	4	1.0 ± 0.2	1.0 ± 0.2	1.0 ± 0.2	1.4 ± 0.2				
	7	0.5 ± 0.1	0.8 ± 0.1	0.6 ± 0.1	0.4 ± 0.1				
Urea (mmol/L)	1	3.3 ± 0.5	3.6 ± 0.5	3.1 ± 0.5	3.3 ± 0.5	0.99	0.55	<0.01	0.02
	2	3.5 ± 0.6	4.3 ± 0.6	5.0 ± 0.6	4.4 ± 0.6				
	4	3.7 ± 0.5	3.9 ± 0.5	3.8 ± 0.5	4.6 ± 0.6				
	7	5.0 ± 0.4	5.4 ± 0.4	4.5 ± 0.4	4.0 ± 0.4				
Non-esterified fatty acids (μmol/L)	1	830 ± 156	709 ± 156	767 ± 156	733 ± 156	0.99	0.70	<0.01	0.95
	2	359 ± 43	368 ± 43	387 ± 43	372 ± 43				
	4	290 ± 71	269 ± 71	226 ± 71	285 ± 75				
	7	152 ± 38	157 ± 40	198 ± 38	164 ± 40				
Triglycerides (mmol/L)	1	0.24 ± 0.04	0.25 ± 0.04	0.26 ± 0.04	0.23 ± 0.04	<0.01	0.63	<0.01	<0.01
	2	0.31 ± 0.04	0.26 ± 0.04	0.30 ± 0.04	0.26 ± 0.04				
	4	0.40 ± 0.06	0.43 ± 0.06	0.21 ± 0.06	0.19 ± 0.07				
	7	0.21 ± 0.02^a^	0.23 ± 0.02^a^	0.10 ± 0.02^b^	0.09 ± 0.02^b^				
Cholesterol (mmol/L)	1	0.62 ± 0.08	0.59 ± 0.08	0.63 ± 0.08	0.60 ± 0.08	0.01	0.90	<0.01	<0.01
	2	0.78 ± 0.07	0.76 ± 0.07	0.92 ± 0.07	0.76 ± 0.07				
	4	1.34 ± 0.09^a^	1.51 ± 0.09^a^	1.00 ± 0.09^b^	0.85 ± 0.09^b^				
	7	1.49 ± 0.12^a^	1.71 ± 0.12^a^	1.03 ± 0.12^b^	0.96 ± 0.12^b^				
Insulin (μg/L)	1	0.59 ± 0.43	0.56 ± 0.45	1.59 ± 0.43	0.96 ± 0.43	0.10	0.21	<0.01	0.43
	2	0.77 ± 0.19	0.89 ± 0.19	1.14 ± 0.19	0.85 ± 0.19				
	4	0.34 ± 0.06	0.29 ± 0.06	0.35 ± 0.06	0.26 ± 0.07				
	7	0.36 ± 0.15	0.39 ± 0.16	0.51 ± 0.15	0.18 ± 0.16				
Glucagon (ng/L)	1	82 ± 17	93 ± 17	120 ± 17	118 ± 17	0.01	1.00	<0.01	<0.01
	2	331 ± 27	324 ± 27	288 ± 27	303 ± 27				
	4	194 ± 15^a^	181 ± 15^ab^	130 ± 15^b^	138 ± 15^ab^				
	7	163 ± 10^a^	159 ± 10^a^	82 ± 10^b^	73 ± 10^b^				
Cortisol (μg/L)	1	36.8 ± 3.7	39.9 ± 3.7	34.3 ± 3.7	42.8 ± 3.7	0.02	0.30	<0.01	0.01
	2	15.6 ± 4.1^ab^	14.3 ± 4.1^b^	28.5 ± 4.1^ab^	31.2 ± 4.1^a^				
	4	13.2 ± 2.7	9.6 ± 2.7	11.5 ± 2.7	15.7 ± 2.8				
	7	9.9 ± 1.3	8.3 ± 1.4	8.1 ± 1.3	11.9 ± 1.4				

^1^Effects: For interactions that are not listed, the probability was *P* > 0.10.

The plasma insulin concentrations decreased with age (*P* < 0.01; Table 2). Glucagon increased to a maximum plasma concentration on day 2 and subsequently decreased in all calves (*P* < 0.01). The glucagon concentration on day 4 and day 7 was higher in the colostrum-fed than in the formula-fed calves (*P* < 0.01; Table 2). The cortisol concentrations decreased with age in all groups (*P* < 0.01) but decreased earlier in the colostrum-fed calves. The mean cortisol concentrations in plasma during the first week of life were lower in the colostrum-fed than in the formula-fed calves (*P* = 0.02; Table 2). Quercetin treatment did not affect the concentrations of metabolites nor hormones in the plasma.

### Immunoglobulins and Acute-Phase Proteins

The plasma concentrations of IgG1 and IgG2 increased on day 2 only in colostrum-fed calves (*P* < 0.01; Fig 3C and 3D). The plasma concentrations of IgM increased sharply until day 2 in colostrum-fed calves and then slowly decreased (*P* < 0.01). In the formula-fed groups, the IgM concentration increased until day 7 (*P* < 0.01) but was still lower than in the colostrum-fed groups (*P* < 0.01) at the end of the trial (Fig 3E). The concentrations of haptoglobin in blood plasma were below the detection limit on day 1 in all calves and increased only in the formula-fed calves (*P* = 0.01) and were highest in ForQ- on day 7 (Fig 3F). The mean haptoglobin concentration was higher in formula-fed than in the colostrum-fed calves (*P* = 0.03). The concentrations of serum amyloid A increased until day 2 and slowly decreased afterwards in all calves (*P* < 0.01; Fig 3G). The concentrations of fibrinogen increased until day 4 and then decreased (*P* < 0.01) without differences among groups (Fig 3H). However, the mean fibrinogen concentration tended to be higher after formula feeding (*P* = 0.10).

### Antioxidative Status

**Fig 4:**
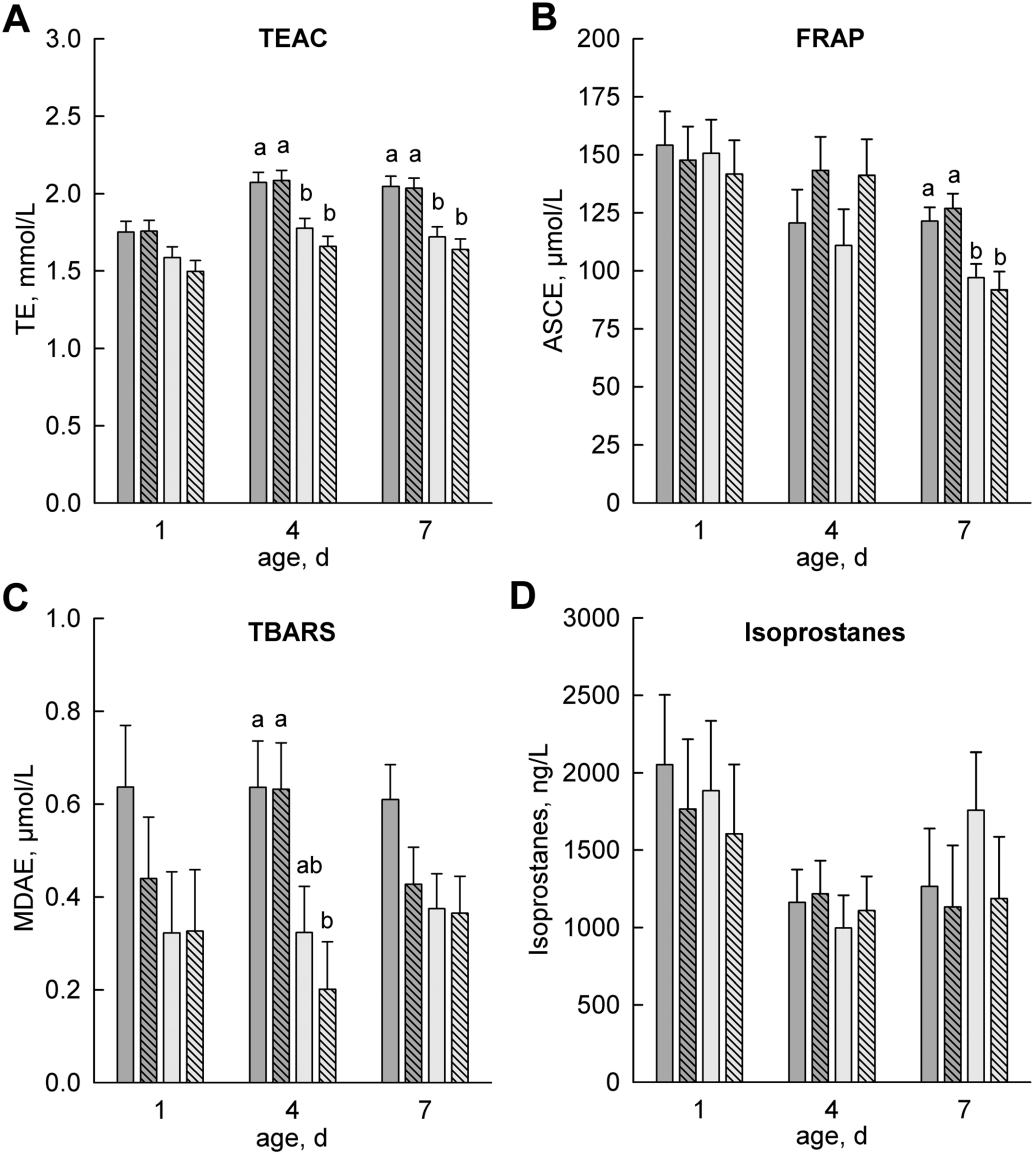
Antioxidative Status in Plasma. Plasma parameters for antioxidative capacity (**A**, **B**) and oxidative stress (**C**, **D**) in calves fed either colostrum (dark grey) or formula (light grey) on days 1 and 2 and supplemented with (crossline) or without (no symbol) quercetin on days 2–8 (50 mg/(kg body weight × day)). Data are presented as the least squares means ± standard errors. Bars with different lowercase letters (a, b) differ among groups within the same day (*P* < 0.05). TEAC, trolox equivalent antioxidative capacity (in trolox equivalents, TE); FRAP, ferric reducing ability of plasma (in ascorbic acid equivalents, ASCE); TBARS, thiobarbituric acid reactive species (in malondialdehyde equivalents, MDAE).

Plasma TEAC increased in all groups until day 4 of life (*P* < 0.01) but was higher in the colostrum-fed than in the formula-fed calves (*P* < 0.01; Fig 4A). FRAP decreased from day 1 to day 7 only in the formula-fed groups (*P* ≤ 0.01) and was higher (*P* < 0.05) on day 7 in the colostrum-fed groups (Fig 4B). Although not statistically significant, the FRAP decrease was delayed in the quercetin-fed calves compared with the control groups. The mean concentrations of TBARS in plasma were higher in the colostrum-fed than in the formula-fed calves (*P* = 0.01) and revealed a diet×age interaction (*P* = 0.01) with higher concentrations in the colostrum-fed than in the formula-fed calves on day 4 (Fig 4C). The mean concentrations of F2-isoprostanes decreased from day 1 to day 4 (*P* < 0.01) in all calves and tended to increase on day 7 only in ForQ- (*P* = 0.08; Fig 4D).

### Hepatic Gene Expression

Regarding inflammation markers, the relative mRNA abundances of *TNF* was higher after quercetin feeding (*P* = 0.04; Table 3). The relative mRNA abundances of *CRP* was higher (*P* = 0.03) and that of *SAA2* tended to be higher (*P* = 0.09) in the formula-fed than in the colostrum-fed groups (Table 3). For the antioxidative enzymes, the mRNA abundances of *CAT* tended to be higher in ColQ- than in ColQ+ (*P* = 0.06) but did not differ between ForQ- and ForQ+, revealing a diet×age interaction (*P* = 0.04; Table 3).

**Table 3:** Hepatic mRNA Expression of Inflammatory and Antioxidative Traits in Calves. Relative mRNA expression of genes related to inflammation and antioxidative status in the liver of 8-d-old calves; data are given in arbitrary units and are presented as the means ± standard errors (SE). Q+, quercetin-supplemented; Q-, control (no quercetin); n = 6 per group. *CAT*, catalase; *GPX1*, glutathione peroxidase; *SOD*, superoxide dismutase; *TNF*, tumor necrosis factor; *IL1A*, interleukin-1α; *IL1B*, interleukin-1β; *HP*, haptoglobin; *FGA*, fibrinogen; *SAA2*, serum amyloid A2; *CRP*, C-reactive protein.

	Group (Diet, Quercetin)					ANOVA *P*-values		
	Colostrum		Formula					
	Q-	Q+	Q-	Q+	SE	Diet	Quercetin	Interaction
**Proinflammatory Cytokines**								
*TNF*	0.98	1.15	0.89	1.48	0.10	0.41	0.04	0.39
*IL1A*	1.17	0.90	1.05	1.18	0.08	0.62	0.66	0.22
*IL1B*	3.00	3.68	3.11	2.69	0.30	0.49	0.83	0.38
**Acute-Phase Proteins**								
*HP*	14.74	29.14	39.78	23.09	4.61	0.30	0.90	0.10
*FGA*	2.73	3.28	3.73	2.18	0.35	0.95	0.48	0.15
*SAA2*	0.91	0.88	1.20	1.12	0.07	0.09	0.72	0.87
*CRP*	0.90	0.96	1.11	1.06	0.04	0.03	0.99	0.42
**Antioxidative Enzymes**								
*CAT*	1.22	0.77	1.01	1.08	0.06	0.68	0.11	0.04
*GPX1*	1.08	0.95	0.98	1.05	0.03	0.99	0.65	0.13
*SOD*	1.03	1.08	1.09	0.90	0.04	0.42	0.36	0.11

## Discussion

In this study, we were able to confirm the oral bioavailability of quercetin in colostrum-fed calves and further showed that after colostrum deprivation, quercetin is absorbed in equal amounts. As indicated earlier, oral bioavailability decreases with age due to intestinal maturation and therefore reduced permeability or more effective elimination processes [24]. The pattern of flavonol fractions in the plasma in this study was different from the patterns in 29-day-old calves or adult cattle but comparable to observations in 2-day-old calves [24, 36]. This result can be explained by the maturation of intestinal metabolism because it has been shown for rats and pigs [37, 38] that orally administered flavonols undergo complete metabolism inside the intestinal mucosa, where glucuronidation is of major importance [39].

Although flavonols were present in the plasma of quercetin-fed calves, we failed to detect any effect on the metabolic parameters or hormone concentrations, which was also the case in previous studies in cattle [24, 36, 40]. However, we could confirm a variety of effects caused by colostrum-deprivation during the first 2 days of life. Cortisol is crucial for initiation of parturition and catabolic activity, especially during hypoxia at birth. However, a decrease of the plasma cortisol concentration is delayed in formula-fed calves, as seen in previous studies [41, 42]. We assume that the delay was possibly caused by either abdominal pain or by reduced utilization of nutrients, which is proven to increase stress parameters in sheep [43, 44].

Higher plasma glucose in colostrum-fed groups was most likely caused by enhanced intestinal maturation and therefore enlarged absorptive surface [45, 46]. Additionally, colostrum feeding seems to accelerate maturation of the pancreas [47] because glucagon concentrations were also higher in colostrum-fed calves from day 4 onwards. Although the basal insulin concentrations were similar among dietary groups, we showed in a companion paper that the postprandial insulin response is more pronounced in colostrum-fed groups [23].

Higher triglyceride and cholesterol concentrations in colostrum-fed calves are in accordance with observations in other studies [41, 42] and are due to the enhanced stimulation of intestinal differentiation by colostral growth factors and hormones. However, it must be considered that diarrhea in formula-fed calves accelerated intestinal transit and therefore reduced absorption time, which might also account for differences in the plasma triglyceride and cholesterol concentrations between dietary groups. Similar concentrations of non-esterified fatty acids indicate that the energy balance in the dietary groups is equal and that lipid mobilization seems to be of minor importance to maintaining energy balance; previous studies also failed to demonstrate consistent effects in neonatal calves [41, 42, 48].

As we expected, the concentration of total protein in the plasma increased only in the colostrum-fed groups, although the protein content of the formula and colostrum was similar. The decrease of the albumin concentration from day 1 to day 2 was probably caused by hemodilution after first feed intake and coincides with previous findings [49, 50]. Because only Ig, but not the albumin concentrations in plasma were affected by colostrum feeding, differences in total protein are most likely caused by absorption of colostral immunoglobulins after colostrum intake [42]. Colostrum intake is important not only for maturational processes but also to acquire passive immunity because the bovine placenta is impermeable to antibodies. As reviewed by Weaver et al. [51], calves with serum IgG concentrations below 10 g/L (total protein < 52 g/L) 24 h after birth suffer from failure of passive transfer, which is associated with increased morbidity and mortality as well as reduced performance. In our study, colostrum-fed calves exceeded this threshold; however, failure of passive transfer was a relevant problem in formula-fed calves, although they were parenterally supplemented with bovine immunoglobulins on day 1. Only formula-fed calves showed slight hyperthermia on the first days of life and a higher incidence of diarrhea, probably caused by gastrointestinal inflammation due to missing local immunity [52], accompanied by abdominal pain and diminished appetite. Obviously, parenteral and oral treatments with immunoglobulins could not prevent local or systemic infections in formula-fed calves, probably because of missing herd-specific immunoglobulins in the formula and the administered drugs. However, we did not perform microbiological analyses in the feces of the calves to determine pathogens that might have caused loose feces. The time course of plasma IgM concentration in formula-fed calves indicates that the indigenous production of IgM is evident as early as day 4 of life, but the concentrations are too low to effectively protect against infections [9].

We assume that inflammatory processes also activated the synthesis of acute phase proteins. Although the increase of the plasma acute phase proteins in all calves emphasized the immunological burden of the new environment, higher haptoglobin concentrations in formula-fed calves suggest more severe inflammatory processes than in colostrum-fed calves [53, 54], which was underlined by the greater incidence of gastrointestinal infections. Because serum amyloid A is more susceptible to stress [53, 55], including physical stress, we suppose that the plasma concentrations were equally high in all groups due to the experimental procedures (e.g., continued venipuncture, restricted feeding, single penning, temporal fixation and dietary changes).

On the mRNA level, we did not find differences in the acute phase proteins between dietary groups because hepatic transcription precedes translation and protein release into circulation. Thus, we obviously missed the time point of elevated mRNA abundances of *HP*. However, mRNA abundance of *CRP*, a moderate acute phase protein, was elevated in the formula-fed groups on day 8, which could indicate the importance of C-reactive protein as a major component of the bovine innate immune system [56]. Hence, increased C-reactive protein production is compensative for the absence of immunoglobulins in colostrum-deprived calves.

Regarding pro-inflammatory cytokines, we found elevated hepatic mRNA abundance of *TNF* in quercetin-supplemented calves but no differences between dietary groups. Under immunocompetent conditions, the impact of various noxae leads to local production of pro-inflammatory cytokines, e.g., tumor necrosis factor (TNF) or interleukin-1, which in turn induce the systemic acute phase response, including synthesis of acute phase proteins [57]. Within signal transduction, reactive oxygen species act as second messengers, thus enhancing TNF-induced gene expression, and oxidative conditions potentiate the activation of respective pathways [57, 58]. Although high doses of quercetin were repeatedly shown to reduce mRNA expression of *TNF in vitro* [59, 60], application of prophylactic doses increased the ratio of pro- to anti-inflammatory cytokines in murine macrophages [60]. We assumed that the hepatic quercetin concentration in our experiment did not exceed the prophylactic dose; thus, quercetin might have increased the expression of *TNF*. Unfortunately, we did not measure TNF protein concentration, but we suppose that posttranscriptional processes anticipated TNF signal transduction [17, 61], because quercetin scavenged reactive oxygen species necessary for signal transduction. Therefore, the observed quercetin effects on *TNF* gene expression could not have been forwarded on the expression of target genes, e.g., *IL1B* or acute phase proteins, as would have been expected otherwise.

Concerning the antioxidative status in the plasma, previous findings in neonatal calves are inconsistent. Inanami et al. [62] and Stohrer, Lutz and Stangassinger [63] concluded from comparisons between calves and dams that the former are highly susceptible to oxidative stress due to immature defense systems, whereas Gaál et al. [64] deduced from high FRAP values, despite the high reactive oxygen species, that calves are well-prepared to address oxidative stress. Although the increase of TEAC in this and a previous study of our group [65] indicates rising antioxidative capacity during the first week of life, this result was not supported by determination of FRAP, another marker of antioxidative capacity. Furthermore, the values of TBARS, which serve as a proxy to measure the products of lipid peroxidation, were unaffected by age in this study, which contradicts earlier studies [62, 64, 66]. However, the reliability of TBARS is criticized for low sensitivity and specificity, and the use of F2-isoprostanes is recommended as the most reliable approach to assess oxidative stress *in vivo* [67, 68]. The time courses of F2-isoprostanes were significantly decreased in all calves; thus, we support the theory that exposure to an oxygen-rich environment following hypoxia during birth results in severe oxidative stress in the newborn [5]. Although quercetin is known to exert antioxidative effects, we did not find improved antioxidative status in the plasma of calves nor increased hepatic expression of antioxidative enzymes, which is in line with previous findings of our group in research conducted on neonatal calves and lactating dairy cows [65, 69]. Orally administered quercetin is completely metabolized inside the intestinal mucosa; thus, the gastrointestinal tract is the first site of action for quercetin [39], as it is for pathogens and gastrointestinal disorders commonly occurring during the first weeks of life. Because we did not examine the intestinal tissue, we cannot exclude local effects of quercetin on antioxidative status. Except for F2-isoprostanes, the parameters of the antioxidative system in the plasma were higher after colostrum feeding. Colostrum is a source of reactive oxygen species, but it also contains a variety of antioxidative factors [66, 70], the latter increasing with time after parturition [71]. The absorption of pro- and antioxidants present in colostrum [72] might have contributed to the rise of the respective parameters in the plasma of colostrum-fed calves and triggered immunological processes, which in turn caused elevated plasma levels. However, the hepatic mRNA abundances of antioxidative enzymes seemed not to be affected by diet, but all groups were fed milk replacer from day 3 onwards and potential dietary effects on hepatic antioxidative status might not be long-lasting.

In conclusion, oral administration of quercetin aglycone at a daily dose of 50 mg/kg body weight to newborn calves during the first week of life is unable to compensate for inadequate colostrum supply. Quercetin did not show any positive effect on neonatal antioxidative or anti-inflammatory status, whereas colostrum feeding improves neonatal health status by supporting passive immunity and by promoting antioxidative/oxidative status.

## Supporting Information

S1 Table Complete data set of parameters regarding dry matter intake and health status as shown in Fig 1. (PDF)

S2 Table Complete data set of flavonoid measurements in blood plasma as shown in Fig 2. (PDF)

S3 Table Complete data set of plasma concentrations of metabolites and hormones as shown in Fig 3 and Table 2. (PDF)

S4 Table Complete data set of immune and inflammatory status in blood plasma as shown in Fig 3. (PDF)

S5 Table Complete data set of antioxidative status in blood plasma as shown in Fig 4. (PDF)

S6 Table Complete data set of hepatic mRNA expression of inflammatory and antioxidative traits as shown in Table 3. (PDF)
