# Supplementary material for: Quercetin Feeding in Newborn Dairy Calves Cannot Compensate Colostrum Deprivation: Study on Metabolic, Antioxidative and Inflammatory Traits
Source: PLoS One. 2016 Jan 11;11(1):e0146932. doi: 10.1371/journal.pone.0146932 (PMC4709053; doi:10.1371/journal.pone.0146932)
Supplement: S1 Table — (PDF) [file pone.0146932.s001.pdf]

calf	group	feeding	quercetin	day of life	body weight (kg)	milk intake, kg/kg body weight	force-fed milk, % of daily intake	fecal score	ctal temp (°	rt rate (per	atory rate (per min)
1	ColQ-	COL	Q-	1	38.5	0.0961	27.027	.	.	.	.
4	ColQ-	COL	Q-	1	46	0.09565	0	.	.	.	.
5	ColQ-	COL	Q-	1	54.5	0.09725	28.302	.	.	.	.
10	ColQ-	COL	Q-	1	46	0.1	0	.	.	.	.
12	ColQ-	COL	Q-	1	44	0.1	0	.	.	.	.
23	ColQ-	COL	Q-	1	48.5	0.10103	0	.	.	.	.
28	ColQ-	COL	Q-	1	39.5	0.10127	0	.	.	.	.
2	ColQ+	COL	Q+	1	44	0.1	0	.	.	.	.
3	ColQ+	COL	Q+	1	52.5	0.09333	0	.	.	.	.
7	ColQ+	COL	Q+	1	42.5	0.09647	0	.	.	.	.
8	ColQ+	COL	Q+	1	50	0.1	0	.	.	.	.
11	ColQ+	COL	Q+	1	43	0.10465	0	.	.	.	.
19	ColQ+	COL	Q+	1	51	0.10588	0	.	.	.	.
26	ColQ+	COL	Q+	1	46	0.1	0	.	.	.	.
9	ForQ-	FOR	Q-	1	51	0.09804	48	.	.	.	.
15	ForQ-	FOR	Q-	1	43	0.1	0	.	.	.	.
18	ForQ-	FOR	Q-	1	44.5	0.09888	13.636	.	.	.	.
21	ForQ-	FOR	Q-	1	45	0.1	37.778	.	.	.	.
22	ForQ-	FOR	Q-	1	37	0.1	18.919	.	.	.	.
24	ForQ-	FOR	Q-	1	48	0.1	0	.	.	.	.
29	ForQ-	FOR	Q-	1	47.5	0.09895	0	.	.	.	.
13	ForQ+	FOR	Q+	1	44.5	0.09888	31.818	.	.	.	.
14	ForQ+	FOR	Q+	1	52	0.1	0	.	.	.	.
17	ForQ+	FOR	Q+	1	43	0.09302	0	.	.	.	.
20	ForQ+	FOR	Q+	1	33	0.1	0	.	.	.	.
25	ForQ+	FOR	Q+	1	46.5	0.10108	0	.	.	.	.
27	ForQ+	FOR	Q+	1	46.5	0.10108	17.021	.	.	.	.
30	ForQ+	FOR	Q+	1	47	0.1	0	.	.	.	.
1	ColQ-	COL	Q-	2	38.5	0.1039	82.5	.	38.7	120	78
4	ColQ-	COL	Q-	2	46	0.12174	0	2	38.4	150	66
5	ColQ-	COL	Q-	2	54.5	0.1156	0	2	38.2	138	56
10	ColQ-	COL	Q-	2	46	0.12174	0	3	38.9	138	66
12	ColQ-	COL	Q-	2	44	0.12045	0	2	38.4	126	48
23	ColQ-	COL	Q-	2	48.5	0.11959	36.207	2	38.9	132	60
28	ColQ-	COL	Q-	2	39.5	0.11899	0	1	38.7	138	48
2	ColQ+	COL	Q+	2	44	0.12273	0	.	38.3	126	48
3	ColQ+	COL	Q+	2	52.5	0.1181	85.484	1	38.5	138	96
7	ColQ+	COL	Q+	2	42.5	0.11765	0	2	38.3	180	48
8	ColQ+	COL	Q+	2	50	0.12	0	2	38.2	150	54
11	ColQ+	COL	Q+	2	43	0.11395	16.327	.	38.6	132	54
19	ColQ+	COL	Q+	2	51	0.11176	0	2	38.5	126	60
26	ColQ+	COL	Q+	2	46	0.12174	0	.	38.5	108	54
9	ForQ-	FOR	Q-	2	51	0.11765	95	.	38.9	162	48
15	ForQ-	FOR	Q-	2	43	0.11628	42	.	38.2	168	60
18	ForQ-	FOR	Q-	2	44.5	0.11685	80.769	3	38.6	144	60
21	ForQ-	FOR	Q-	2	45	0.11778	100	2	38.9	132	66
22	ForQ-	FOR	Q-	2	37	0.12162	100	2.75	38.6	144	54
24	ForQ-	FOR	Q-	2	48	0.12083	29.31	3	38.9	138	48
29	ForQ-	FOR	Q-	2	47.5	0.11579	18.182	3	38.3	120	36
13	ForQ+	FOR	Q+	2	44.5	0.11011	0	.	39.5	166	48

14	ForQ+	FOR	Q+	2	52	0.10962	26.316	2	39.4	126	66
17	ForQ+	FOR	Q+	2	43	0.11395	0	2.5	38.9	138	48
20	ForQ+	FOR	Q+	2	33	0.12424	95.122	2	38.1	156	72
25	ForQ+	FOR	Q+	2	46.5	0.11613	88.889	2.5	38.7	132	72
27	ForQ+	FOR	Q+	2	46.5	0.12043	100	3	38.9	168	60
30	ForQ+	FOR	Q+	2	47	0.11915	103.571	2	38.4	114	60
1	ColQ-	COL	Q-	3	40	0.115	0	2	38.8	108	66
4	ColQ-	COL	Q-	3	46	0.12174	0	2	38.7	132	60
5	ColQ-	COL	Q-	3	56	0.11786	0	1	38.8	126	54
10	ColQ-	COL	Q-	3	48.5	0.11753	0	3	39.4	150	90
12	ColQ-	COL	Q-	3	46	0.12391	0	2	39.1	126	66
23	ColQ-	COL	Q-	3	50.5	0.10693	20.37	2	39	108	48
28	ColQ-	COL	Q-	3	39.5	0.12405	0	2	39.1	180	48
2	ColQ+	COL	Q+	3	46.5	0.11613	0	2	38.8	72	.
3	ColQ+	COL	Q+	3	55	0.09273	19.608	1	38.6	126	90
7	ColQ+	COL	Q+	3	44	0.11818	0	.	.	.	.
8	ColQ+	COL	Q+	3	52	0.11923	0	.	38.7	132	54
11	ColQ+	COL	Q+	3	45	0.10889	38.776	3	39.2	120	42
19	ColQ+	COL	Q+	3	52.5	0.08	23.81	2	38.6	138	42
26	ColQ+	COL	Q+	3	47	0.11915	0	.	39.6	132	66
9	ForQ-	FOR	Q-	3	51	0.11765	91.667	3	39.4	144	48
15	ForQ-	FOR	Q-	3	45.5	0.11868	0	2	38.8	132	36
18	ForQ-	FOR	Q-	3	45.5	0.1011	0	3	38.7	174	72
21	ForQ-	FOR	Q-	3	48	0.12083	0	2	39.5	120	54
22	ForQ-	FOR	Q-	3	38	0.12105	0	2.75	38.8	114	66
24	ForQ-	FOR	Q-	3	48	0.06042	86.207	2.5	39.4	138	.
29	ForQ-	FOR	Q-	3	50.5	0.06535	0	3	39.2	96	39
13	ForQ+	FOR	Q+	3	47.5	0.08421	17.5	2	39.3	168	48
14	ForQ+	FOR	Q+	3	53	0.11698	0	2	39.2	126	54
17	ForQ+	FOR	Q+	3	44.5	0.11685	0	2	39.7	138	42
20	ForQ+	FOR	Q+	3	34.5	0.08986	16.129	2	38.6	162	90
25	ForQ+	FOR	Q+	3	48	0.12083	0	2.5	39.4	126	72
27	ForQ+	FOR	Q+	3	44	0.01364	0	4	38.6	144	48
30	ForQ+	FOR	Q+	3	45.5	0.06593	0	3	39.1	114	66
1	ColQ-	COL	Q-	4	39.25	0.11849	0	2	39.2	120	102
4	ColQ-	COL	Q-	4	46	0.12174	0	.	38.4	138	54
5	ColQ-	COL	Q-	4	56	0.11786	0	2	39.2	.	.
10	ColQ-	COL	Q-	4	48.5	0.11959	0	2	39.4	168	102
12	ColQ-	COL	Q-	4	46	0.11957	0	2	39.4	162	72
23	ColQ-	COL	Q-	4	49.5	0.08233	0	2	38.5	132	48
28	ColQ-	COL	Q-	4	39.5	0.12405	0	2	39.1	180	33
2	ColQ+	COL	Q+	4	46.5	0.12043	0	2	39.3	102	66
3	ColQ+	COL	Q+	4	53.75	0.11996	37.903	1	39	156	84
7	ColQ+	COL	Q+	4	44	0.11818	0	1	38.7	162	78
8	ColQ+	COL	Q+	4	51	0.12162	0	.	.	.	.
11	ColQ+	COL	Q+	4	45	0.12	0	2	38.9	114	42
19	ColQ+	COL	Q+	4	52.5	0.1181	0	2	38.5	108	30
26	ColQ+	COL	Q+	4	46.5	0.11936	0	1	39.2	126	66
9	ForQ-	FOR	Q-	4	51	0.04706	0	3	39.4	120	48
15	ForQ-	FOR	Q-	4	44.25	0.06837	0	.	38.9	150	42
18	ForQ-	FOR	Q-	4	45	0.04948	0	2	38.9	.	.

21	ForQ-	FOR	Q-	4	46.5	0.08153	0	2	39	114	60
22	ForQ-	FOR	Q-	4	37.5	0.07945	0	2	39.5	108	60
24	ForQ-	FOR	Q-	4	48	0.12083	0	.	39.2	114	54
29	ForQ-	FOR	Q-	4	50.5	0.08515	0	.	38.6	108	48
13	ForQ+	FOR	Q+	4	47.5	0.12	0	2	39.4	126	30
14	ForQ+	FOR	Q+	4	52.5	0.09022	0	.	39.1	138	66
17	ForQ+	FOR	Q+	4	44.5	0.11685	0	2	39.4	150	54
20	ForQ+	FOR	Q+	4	34.5	0.13333	0	2	39.4	168	84
25	ForQ+	FOR	Q+	4	47.25	0.02718	0	2	39	132	78
27	ForQ+	FOR	Q+	4	44	0.02045	0	3	38.7	.	.
30	ForQ+	FOR	Q+	4	46.25	0.06218	0	2	39.2	114	66
1	ColQ-	COL	Q-	5	40	0.12	0	.	38.9	116	72
4	ColQ-	COL	Q-	5	46	0.12174	0	.	37.6	138	42
5	ColQ-	COL	Q-	5	56	0.11786	0	2	39.1	132	42
10	ColQ-	COL	Q-	5	48.5	0.11959	0	2	39.1	156	54
12	ColQ-	COL	Q-	5	46	0.12174	0	.	38.2	.	.
23	ColQ-	COL	Q-	5	50.5	0.11881	0	2	38.9	120	60
28	ColQ-	COL	Q-	5	39.5	0.12658	0	2	38.9	156	.
2	ColQ+	COL	Q+	5	46.5	0.12043	0	2	39.1	102	54
3	ColQ+	COL	Q+	5	55	0.12	0	2	38.9	138	84
7	ColQ+	COL	Q+	5	44	0.12045	0	2	38.8	162	.
8	ColQ+	COL	Q+	5	52	0.11923	0	2	37.7	120	42
11	ColQ+	COL	Q+	5	45	0.12	0	.	39	138	54
19	ColQ+	COL	Q+	5	52.5	0.1181	0	2	38.8	132	30
26	ColQ+	COL	Q+	5	47	0.11915	0	1	39	138	66
9	ForQ-	FOR	Q-	5	51	0.04706	0	4	39	114	42
15	ForQ-	FOR	Q-	5	45.5	0.12088	0	2	39.1	162	42
18	ForQ-	FOR	Q-	5	45.5	0.07253	0	.	.	.	.
21	ForQ-	FOR	Q-	5	48	0.12083	0	2	39.4	120	48
22	ForQ-	FOR	Q-	5	38	0.12105	0	2	38.8	132	60
24	ForQ-	FOR	Q-	5	48	0.12083	0	.	39.3	108	60
29	ForQ-	FOR	Q-	5	50.5	0.11485	0	.	38.7	102	36
13	ForQ+	FOR	Q+	5	47.5	0.12	0	.	38.8	126	30
14	ForQ+	FOR	Q+	5	53	0.12075	0	2	39.7	132	54
17	ForQ+	FOR	Q+	5	44.5	0.12135	0	2	38.7	144	.
20	ForQ+	FOR	Q+	5	34.5	0.14493	0	2	38.6	148	76
25	ForQ+	FOR	Q+	5	48	0.12083	0	2	38.8	138	60
27	ForQ+	FOR	Q+	5	44	0.14091	0	3	38.4	114	30
30	ForQ+	FOR	Q+	5	45.5	0.06154	0	.	.	.	.
1	ColQ-	COL	Q-	6	40	0.12	0	.	38.1	126	84
4	ColQ-	COL	Q-	6	46	0.12174	0	.	38.1	.	.
5	ColQ-	COL	Q-	6	56	0.11964	0	.	38.3	168	54
10	ColQ-	COL	Q-	6	48.5	0.12371	0	2	.	.	.
12	ColQ-	COL	Q-	6	46	0.12174	0	2	39.1	126	126
23	ColQ-	COL	Q-	6	50.5	0.11881	0	.	38.7	108	60
28	ColQ-	COL	Q-	6	39.5	0.12658	0	2	38.7	186	.
2	ColQ+	COL	Q+	6	46.5	0.12043	0	.	38.9	126	.
3	ColQ+	COL	Q+	6	55	0.12364	0	2	38.5	126	78
7	ColQ+	COL	Q+	6	44	0.12045	0	.	38.3	180	36
8	ColQ+	COL	Q+	6	52	0.12115	0	.	38.3	150	42
11	ColQ+	COL	Q+	6	45	0.12222	0	2	38.1	144	48

19	ColQ+	COL	Q+	6	52.5	0.12	0	.	38.9	120	48
26	ColQ+	COL	Q+	6	47	0.11915	0	2	39.4	132	72
9	ForQ-	FOR	Q-	6	51	0.06078	0	4	38.7	90	30
15	ForQ-	FOR	Q-	6	45.5	0.11868	0	.	38.8	.	.
18	ForQ-	FOR	Q-	6	45.5	0.1033	0	3	39.2	108	.
21	ForQ-	FOR	Q-	6	48	0.12083	0	2	38.9	132	54
22	ForQ-	FOR	Q-	6	38	0.1	0	2	39.1	126	48
24	ForQ-	FOR	Q-	6	48	0.12083	0	.	39.1	126	54
29	ForQ-	FOR	Q-	6	50.5	0.11881	0	3	39.6	96	42
13	ForQ+	FOR	Q+	6	47.5	0.12	0	3	38.9	108	.
14	ForQ+	FOR	Q+	6	53	0.12075	0	.	39.2	132	60
17	ForQ+	FOR	Q+	6	44.5	0.12135	0	2	38.7	132	36
20	ForQ+	FOR	Q+	6	34.5	0.12464	0	2	39.1	126	84
25	ForQ+	FOR	Q+	6	48	0.12083	0	2	38.5	156	72
27	ForQ+	FOR	Q+	6	45	0.12	0	.	38.7	102	30
30	ForQ+	FOR	Q+	6	45.5	0.12088	0	2	39.4	132	.
1	ColQ-	COL	Q-	7	40	0.1125	0	2	38.9	116	78
4	ColQ-	COL	Q-	7	46.5	0.11613	0	2	38.7	116	.
5	ColQ-	COL	Q-	7	57.5	0.10087	0	2	37.6	114	.
10	ColQ-	COL	Q-	7	51	0.10784	0	.	38.6	126	42
12	ColQ-	COL	Q-	7	48	0.11042	0	.	38.3	138	138
23	ColQ-	COL	Q-	7	52	0.10962	0	.	39.2	114	84
28	ColQ-	COL	Q-	7	41	0.10976	0	2	.	204	.
2	ColQ+	COL	Q+	7	47.5	0.12	0	.	38.3	96	54
3	ColQ+	COL	Q+	7	56.5	0.12035	0	.	38.9	126	66
7	ColQ+	COL	Q+	7	45.5	0.10989	0	.	.	.	.
8	ColQ+	COL	Q+	7	52.5	.	.	.	.	.	.
11	ColQ+	COL	Q+	7	46.5	0.11183	0	.	38.7	126	36
19	ColQ+	COL	Q+	7	53.5	0.10654	0	2	39.1	.	.
26	ColQ+	COL	Q+	7	49	0.1102	0	.	39.2	120	60
9	ForQ-	FOR	Q-	7	50	0.1	0	4	39.1	96	36
15	ForQ-	FOR	Q-	7	46.5	0.10968	0	.	38.5	162	.
18	ForQ-	FOR	Q-	7	43.5	0.11264	0	3	39.2	108	.
21	ForQ-	FOR	Q-	7	51	0.10588	0	2	38.6	138	54
22	ForQ-	FOR	Q-	7	38	0.11316	0	2.5	38.9	126	42
24	ForQ-	FOR	Q-	7	48	0.11042	0	2	39.3	120	48
29	ForQ-	FOR	Q-	7	52	0.06538	0	2	40	96	42
13	ForQ+	FOR	Q+	7	51	0.10784	0	.	39.1	.	.
14	ForQ+	FOR	Q+	7	55	0.10545	0	3	39.5	132	60
17	ForQ+	FOR	Q+	7	47	0.10851	0	3	38.6	.	.
20	ForQ+	FOR	Q+	7	36	0.10833	0	3	38.9	114	60
25	ForQ+	FOR	Q+	7	47.5	0.10947	0	.	.	.	.
27	ForQ+	FOR	Q+	7	45	0.11111	0	.	38.6	108	36
30	ForQ+	FOR	Q+	7	42.5	.	.	2	39.3	102	.
1	ColQ-	COL	Q-	8	.	.	.	.	38.6	104	60
4	ColQ-	COL	Q-	8	.	.	.	.	38.9	120	36
5	ColQ-	COL	Q-	8	.	.	.	3	38.8	.	.
10	ColQ-	COL	Q-	8	.	.	.	2	38.9	.	.
12	ColQ-	COL	Q-	8	.	.	.	2	39	126	126
23	ColQ-	COL	Q-	8	.	.	.	2	38.8	126	42
28	ColQ-	COL	Q-	8	.	.	.	.	38.8	162	.

2	ColQ+	COL	Q+	8	.	.	.		2	38.7	.	
3	ColQ+	COL	Q+	8	.	.	.		2	38.8	114	.
7	ColQ+	COL	Q+	8	.	.	.		2	38.5	96	24
8	ColQ+	COL	Q+	8	.	.	.	.		38.9	.	.
11	ColQ+	COL	Q+	8	.	.	.	.		38.6	120	30
19	ColQ+	COL	Q+	8	.	.	.		2	39.1	120	.
26	ColQ+	COL	Q+	8	.	.	.		2	38.7	150	42
9	ForQ-	FOR	Q-	8	.	.	.		3	.	.	.
15	ForQ-	FOR	Q-	8	.	.	.	.	.	.	.	
18	ForQ-	FOR	Q-	8	.	.	.		3	39	102	.
21	ForQ-	FOR	Q-	8	.	.	.		2	38.7	102	51
22	ForQ-	FOR	Q-	8	.	.	.		3	38.3	108	48
24	ForQ-	FOR	Q-	8	.	.	.	.	.	.	.	
29	ForQ-	FOR	Q-	8	.	.	.	.	.	.	.	
13	ForQ+	FOR	Q+	8	.	.	.		3	39.1	.	.
14	ForQ+	FOR	Q+	8	.	.	.		2	39.2	132	54
17	ForQ+	FOR	Q+	8	.	.	.	.		38.8	132	.
20	ForQ+	FOR	Q+	8	.	.	.		3	38.9	.	.
25	ForQ+	FOR	Q+	8	.	.	.		2	38.9	114	48
27	ForQ+	FOR	Q+	8	.	.	.		2	39.1	132	42
30	ForQ+	FOR	Q+	8	.	.	.		3.5	39.6	.	.
