# Supplementary material for: Quercetin Feeding in Newborn Dairy Calves Cannot Compensate Colostrum Deprivation: Study on Metabolic, Antioxidative and Inflammatory Traits
Source: PLoS One. 2016 Jan 11;11(1):e0146932. doi: 10.1371/journal.pone.0146932 (PMC4709053; doi:10.1371/journal.pone.0146932)
Supplement: S2 Table — (PDF) [file pone.0146932.s002.pdf]

calf	identification	group	feeding	quercetin	day of life	Quercetin, nmol/L	Isorhamnetin, nmol/L	Tamarixetin, nmol/L	Kaempferol, nmol/L	sum, nmol/L	Q_percent	I_percent	T_percent	K_percent	Ratio_KIT_Q
2	44173	CQ+	COL	yes	1	7.85	0	0	3.9759	11.826	66.381	0	0	33.6187	0.50645
3	96015	CQ+	COL	yes	1	7.969	0	0	4.0806	12.05	66.136	0	0	33.8638	0.51203
7	17503	CQ+	COL	yes	1	0	0	0	0	0	0	0	0	0	0
8	17507	CQ+	COL	yes	1	0	0	0	0	0	0	0	0	0	0
11	96108	CQ+	COL	yes	1	0	0	0	0	0	0	0	0	0	0
13	96121	FQ+	FOR	yes	1	0	0	0	0	0	0	0	0	0	0
14	96124	FQ+	FOR	yes	1	0	0	0	0	0	0	0	0	0	0
17	96138	FQ+	FOR	yes	1	0	0	0	0	0	0	0	0	0	0
19	96149	CQ+	COL	yes	1	0	0	0	0	0	0	0	0	0	0
20	17627	FQ+	FOR	yes	1	0	0	0	0	0	0	0	0	0	0
25	17639	FQ+	FOR	yes	1	0	0	0	0	0	0	0	0	0	0
26	96185	CQ+	COL	yes	1	0	0	0	0	0	0	0	0	0	0
27	17642	FQ+	FOR	yes	1	0	0	0	0	0	0	0	0	0	0
30	17647	FQ+	FOR	yes	1	0	0	0	0	0	0	0	0	0	0
2	44173	CQ+	COL	yes	2	6.606	2.098	0	4.0518	12.756	51.789	16.4465	0	31.7649	0.93092
3	96015	CQ+	COL	yes	2	8.006	5.358	5.213	0	18.577	43.098	28.8405	28.0613	0	1.32028
7	17503	CQ+	COL	yes	2	0	0	0	0	0	0	0	0	0	0
8	17507	CQ+	COL	yes	2	0	0	0	0	0	0	0	0	0	0
11	96108	CQ+	COL	yes	2	0	0	0	0	0	0	0	0	0	0
13	96121	FQ+	FOR	yes	2	0	0	0	0	0	0	0	0	0	0
14	96124	FQ+	FOR	yes	2	0	0	0	0	0	0	0	0	0	0
17	96138	FQ+	FOR	yes	2	0	0	0	0	0	0	0	0	0	0
19	96149	CQ+	COL	yes	2	0	0	0	0	0	0	0	0	0	0
20	17627	FQ+	FOR	yes	2	0	0	0	0	0	0	0	0	0	0
25	17639	FQ+	FOR	yes	2	0	0	0	0	0	0	0	0	0	0
26	96185	CQ+	COL	yes	2	0	0	0	0	0	0	0	0	0	0
27	17642	FQ+	FOR	yes	2	0	0	0	0	0	0	0	0	0	0
30	17647	FQ+	FOR	yes	2	0	0	0	0	0	0	0	0	0	0
2	44173	CQ+	COL	yes	3	.	.	.	.	.	.	.	.	.	.
3	96015	CQ+	COL	yes	3	62.32	59.12	30	0	151.44	41.152	39.0386	19.8098	0	1.43004
7	17503	CQ+	COL	yes	3	442.07	164.64	173.79	15.75	796.25	55.519	20.6769	21.8261	1.978	0.80119
8	17507	CQ+	COL	yes	3	146.25	56.897	54.945	8.7674	266.859	54.804	21.321	20.5896	3.2854	0.82469
11	96108	CQ+	COL	yes	3	251.482	50.674	43.272	12.1299	357.559	70.333	14.1723	12.1022	3.3924	0.42181
13	96121	FQ+	FOR	yes	3	338.903	85.44	88.522	12.8381	525.703	64.467	16.2525	16.8389	2.4421	0.55119
14	96124	FQ+	FOR	yes	3	287.612	57.044	33.413	14.0687	392.138	73.345	14.547	8.5207	3.5877	0.36343
17	96138	FQ+	FOR	yes	3	.	.	.	.	.	.	.	.	.	.
19	96149	CQ+	COL	yes	3	.	.	.	.	.	.	.	.	.	.
20	17627	FQ+	FOR	yes	3	.	.	.	.	.	.	.	.	.	.
25	17639	FQ+	FOR	yes	3	24.192	19.666	8.65	2.7849	55.293	43.753	35.5671	15.6434	5.0367	1.28557
26	96185	CQ+	COL	yes	3	96.844	34.327	14.712	2.9816	148.865	65.055	23.0594	9.8831	2.0029	0.53717
27	17642	FQ+	FOR	yes	3	167.896	32.723	18.219	5.8081	224.646	74.738	14.5666	8.11	2.5854	0.33801
30	17647	FQ+	FOR	yes	3	96.733	23.013	21.926	3.5314	145.204	66.619	15.849	15.1	2.4321	0.50108
2	44173	CQ+	COL	yes	4	248.16	94.744	56.899	13.4129	413.215	60.056	22.9284	13.7697	3.246	0.66512
3	96015	CQ+	COL	yes	4	58.998	30.973	24.604	7.8548	122.43	48.189	25.2984	20.0967	6.4158	1.07516
7	17503	CQ+	COL	yes	4	142.42	69.31	29.24	0	240.97	59.103	28.7629	12.1343	0	0.69197
8	17507	CQ+	COL	yes	4	143.141	64.269	51.01	7.3421	265.762	53.861	24.183	19.1938	2.7627	0.85665
11	96108	CQ+	COL	yes	4	130.985	38.251	20.963	7.3405	197.539	66.308	19.3636	10.6121	3.716	0.50811
13	96121	FQ+	FOR	yes	4	.	.	.	.	.	.	.	.	.	.
14	96124	FQ+	FOR	yes	4	87.991	21.646	13.398	4.8179	127.853	68.822	16.9306	10.4794	3.7683	0.45303
17	96138	FQ+	FOR	yes	4	35.987	12.32	8.637	3.2762	60.221	59.759	20.4581	14.3427	5.4404	0.67339
19	96149	CQ+	COL	yes	4	93.096	47.86	30.393	5.6078	176.956	52.609	27.0463	17.1753	3.169	0.9008
20	17627	FQ+	FOR	yes	4	37.224	15.659	2.205	0	55.088	67.572	28.4259	4.002	0	0.4799

25	17639	FQ+	FOR	yes	4	69.207	22.767	9.919	2.0801	103.973	66.563	21.8965	9.5401	2.0007	0.50234
26	96185	CQ+	COL	yes	4	50.863	18.959	0	0	69.822	72.846	27.1538	0	0	0.37276
30	17647	FQ+	FOR	yes	4	91.096	20.46	31.805	3.105	146.466	62.196	13.9689	21.7152	2.1199	0.60782
2	44173	CQ+	COL	yes	7	49.054	20.021	9.63	6.1586	84.864	57.803	23.5922	11.3476	7.2571	0.73001
3	96015	CQ+	COL	yes	7	37.108	21.098	14.833	2.1688	75.209	49.341	28.0527	19.7229	2.8837	1.02672
7	17503	CQ+	COL	yes	7	114.75	30.36	22.76	3.45	171.32	66.98	17.7212	13.2851	2.0138	0.49298
11	96108	CQ+	COL	yes	7	82.213	31.798	30.46	6.4607	150.931	54.47	21.0676	20.1815	4.2806	0.83586
13	96121	FQ+	FOR	yes	7	133.499	29.247	33.533	4.4738	200.752	66.499	14.5685	16.7037	2.2285	0.50377
14	96124	FQ+	FOR	yes	7	103.231	14.519	12.442	5.0035	135.196	76.357	10.7396	9.2027	3.7009	0.30964
17	96138	FQ+	FOR	yes	7	16.76	5.331	4.908	2.072	29.07	57.651	18.3365	16.8846	7.1274	0.73456
19	96149	CQ+	COL	yes	7	151.937	54.495	52.604	7.8448	266.88	56.931	20.4192	19.7106	2.9395	0.75652
20	17627	FQ+	FOR	yes	7	107.243	22.783	15.851	2.6877	148.564	72.186	15.3352	10.6695	1.8091	0.38531
25	17639	FQ+	FOR	yes	7	50.308	23.449	20.763	3.2455	97.765	51.458	23.9847	21.238	3.3197	0.94335
26	96185	CQ+	COL	yes	7	30.657	0	0	0	30.657	100	0	0	0	0
27	17642	FQ+	FOR	yes	7	62.657	14.244	18.096	3.1707	98.167	63.826	14.5099	18.4337	3.2299	0.56675
2	44173	CQ+	COL	yes	8	63.343	19.154	9.372	4.6322	96.501	65.639	19.8488	9.7117	4.8001	0.52348
3	96015	CQ+	COL	yes	8	.	.	.	.	.	.	.	.	.	.
7	17503	CQ+	COL	yes	8	.	.	.	.	.	.	.	.	.	.
11	96108	CQ+	COL	yes	8	.	.	.	.	.	.	.	.	.	.
13	96121	FQ+	FOR	yes	8	161.704	19.12	16.33	5.0544	202.208	79.969	9.4555	8.0757	2.4996	0.25048
14	96124	FQ+	FOR	yes	8	89.841	10.458	6.72	3.9153	110.935	80.986	9.4272	6.0578	3.5294	0.23479
17	96138	FQ+	FOR	yes	8	.	.	.	.	.	.	.	.	.	.
19	96149	CQ+	COL	yes	8	74.96	16.3	35.961	5.1305	132.351	56.637	12.316	27.1706	3.8764	0.76563
20	17627	FQ+	FOR	yes	8	76.688	14.205	11.083	29.195	131.17	58.464	10.8292	8.4493	22.2573	0.71045
25	17639	FQ+	FOR	yes	8	43.227	9.308	13.496	2.47	68.5	63.105	13.5877	19.7017	3.6058	0.58466
26	96185	CQ+	COL	yes	8	100.191	11.644	15.68	2.0032	129.518	77.357	8.99	12.1067	1.5467	0.29271
27	17642	FQ+	FOR	yes	8	7.801	6.937	9.782	.	24.52	31.816	28.2917	39.8922	.	.
