# Supplementary material for: Quercetin Feeding in Newborn Dairy Calves Cannot Compensate Colostrum Deprivation: Study on Metabolic, Antioxidative and Inflammatory Traits
Source: PLoS One. 2016 Jan 11;11(1):e0146932. doi: 10.1371/journal.pone.0146932 (PMC4709053; doi:10.1371/journal.pone.0146932)
Supplement: S3 Table — (PDF) [file pone.0146932.s003.pdf]

calf	group	feeding	quercetin	day of life	NEFA, µmol/L	urea, mmol/L	triglycerides, mmol/L	Lactate, mmol/L	Glucose, mmol/L	Cholesterol, mmol/L	Albumin, g/L	Protein, g/L	Cortisol, µg/L	Insulin, µg/L	Glucagon, ng/L
1	ColQ-	COL	Q-	1	895	4.27	0.23	2.57	2.62	0.39	26.1	45.3	29.9	0.42164	74.45
4	ColQ-	COL	Q-	1	1130	3.24	0.23	1.78	3.62	0.41	24.7	42.4	28.8	0.27239	89.945
5	ColQ-	COL	Q-	1	1156	3	0.24	1.92	4.27	0.82	24	41.6	39.6	0.52239	71.655
10	ColQ-	COL	Q-	1	223	4.25	0.25	7.68	6.02	0.96	24.9	44	32	1.47948	83.6
12	ColQ-	COL	Q-	1	1393	3.14	0.36	2.96	6.07	0.59	28.4	47.3	38.4	0.92351	78.35
23	ColQ-	COL	Q-	1	368	2.47	0.16	6.06	3.39	0.69	26.4	45.4	57.6	0.24254	97.3
28	ColQ-	COL	Q-	1	646	2.79	0.22	5.29	3.81	0.45	26.1	45.8	31.6	0.23507	80
2	ColQ+	COL	Q+	1	566	3.39	0.18	2.28	4.35	0.73	25.9	46.7	39.4	0.48694	57.25
3	ColQ+	COL	Q+	1	1478	2.89	0.33	2.2	2.97	0.53	25.8	44.5	44.1	0.375	66.65
7	ColQ+	COL	Q+	1	626	3.28	0.14	4.27	6.89	0.52	26.1	46.9	46.9	0.21269	104.3
8	ColQ+	COL	Q+	1	46	6.99	0.49	.	7.67	0.42	27.5	47.9	23.8	.	187
11	ColQ+	COL	Q+	1	500	2.6	0.16	3.02	5.08	0.36	27.8	48.8	40.2	1.73507	62.35
19	ColQ+	COL	Q+	1	1070	3.1	0.32	2.91	2.72	0.64	26.7	47.9	43.2	0.54664	95
26	ColQ+	COL	Q+	1	679	3.13	0.15	2.28	3.48	0.94	26.2	45	41.8	0.13993	80.85
9	ForQ-	FOR	Q-	1	321	5.96	0.45	8.11	2.63	0.59	23.7	49.3	44.7	0.52425	260.9
15	ForQ-	FOR	Q-	1	1597	3.21	0.42	1.67	5.13	0.85	27.2	50	19.8	0.41978	57.9
18	ForQ-	FOR	Q-	1	308	2.65	0.22	6.31	5	0.48	24.2	42.3	34.9	3.35261	97.45
21	ForQ-	FOR	Q-	1	382	2.84	0.14	2.9	2.72	0.84	27.3	45.9	38.9	0.32649	93.95
22	ForQ-	FOR	Q-	1	950	1.34	0.19	2.555	2.885	0.66	24.4	41.35	32.2	0.54851	127.4
24	ForQ-	FOR	Q-	1	966	2.72	0.19	2.9	6.28	0.68	27.8	46	29.8	5.45709	127.75
29	ForQ-	FOR	Q-	1	844	3.27	0.21	1.75	3.83	0.29	25.3	44.2	39.5	0.49627	74.25
13	ForQ+	FOR	Q+	1	565	2.93	0.27	2.72	5.16	0.39	26.3	43.5	27.6	0.31343	63.3
14	ForQ+	FOR	Q+	1	448	3.85	0.18	3.25	3.54	0.58	24.2	42.3	44.6	1.13806	85.25
17	ForQ+	FOR	Q+	1	659	2.65	0.29	3.38	3.57	0.54	26.9	47.3	38.5	1.13806	121.2
20	ForQ+	FOR	Q+	1	1160	3.31	0.32	4.01	6.51	0.78	26.5	47.3	46.9	1.59328	146.65
25	ForQ+	FOR	Q+	1	514	2.57	0.11	3.35	3.85	0.85	24.6	43.4	67.6	0.20149	136.95
27	ForQ+	FOR	Q+	1	833	5.29	0.18	2.84	3.63	0.4	24.8	43.5	38.9	0.66045	88.55
30	ForQ+	FOR	Q+	1	952	2.57	0.23	2.45	2.52	0.67	28.7	47.4	35.7	1.65485	181.6
1	ColQ-	COL	Q-	2	233	4.11	0.2	1.43	5.8	0.84	22.7	65.7	11.2	0.94963	400
4	ColQ-	COL	Q-	2	389	3.64	0.26	2.33	6.13	0.52	23.6	56.3	10	0.4403	398.8
5	ColQ-	COL	Q-	2	199	3.41	0.27	2.8	5.35	0.96	21.2	58.8	15.6	0.93843	241.35
10	ColQ-	COL	Q-	2	399	4.4	0.36	1.94	6.31	1.11	22.2	63.2	8.1	0.62127	227.6
12	ColQ-	COL	Q-	2	402	3.3	0.24	2.85	7	0.61	23.9	55	23.4	1.47575	308.5
23	ColQ-	COL	Q-	2	609	3.51	0.4	2.69	5.61	0.82	23.5	63	27.3	0.63806	341.5
28	ColQ-	COL	Q-	2	279	2.21	0.47	2.9	5.46	0.57	23.4	55.9	13.7	0.33955	400
2	ColQ+	COL	Q+	2	348	5.08	0.24	4.13	5.63	0.87	23.3	70.4	10.1	0.375	313.4
3	ColQ+	COL	Q+	2	410	3.82	0.47	2.85	5.08	0.65	22.1	71.2	9.6	1.38246	399.85
7	ColQ+	COL	Q+	2	390	3.71	0.17	3.35	5.87	0.63	23.2	62.8	30.9	0.54291	333.15
8	ColQ+	COL	Q+	2	554	6.49	0.32	2.47	5.27	0.87	24.6	60.7	6.5	1.33396	400
11	ColQ+	COL	Q+	2	293	3.83	0.24	3.75	6.57	0.68	24.7	62.3	16.6	1.5	346.15
19	ColQ+	COL	Q+	2	174	4.05	0.23	2.37	5.43	0.73	23.7	58	14.2	0.93843	265
26	ColQ+	COL	Q+	2	410	3.09	0.16	1.96	4.82	0.87	23.4	57.1	12	0.18097	208.95
9	ForQ-	FOR	Q-	2	314	10.71	0.57	4	7.01	1.21	23	51.4	46.5	1.54104	363.65
15	ForQ-	FOR	Q-	2	535	4.37	0.2	2.43	6.85	0.92	25.2	48.2	19	1.79851	317.2
18	ForQ-	FOR	Q-	2	366	3.57	0.29	2.89	3.59	1.05	23.7	44.3	18	0.86567	323
21	ForQ-	FOR	Q-	2	370	6	0.4	3.22	4.96	1.08	26.3	47.4	32.2	0.59888	251.6
22	ForQ-	FOR	Q-	2	566	2.43	0.21	1.43	4.93	0.86	23.5	40.8	45.4	0.26679	266.25
24	ForQ-	FOR	Q-	2	295	3.49	0.14	3.09	7.45	0.74	25	45.9	16.5	1.07463	304.95
29	ForQ-	FOR	Q-	2	262	4.32	0.28	1.67	5.94	0.55	23	42.4	21.6	1.84515	187.25
13	ForQ+	FOR	Q+	2	368	6.26	0.24	1.49	4.86	0.53	25.3	45.6	29.6	0.58582	249.75
14	ForQ+	FOR	Q+	2	309	4.77	0.25	2.06	7.12	0.76	23.1	41.8	27.6	1.42537	175.9
17	ForQ+	FOR	Q+	2	345	5.29	0.41	2.89	5.7	0.89	25.6	47.1	14.7	1.03545	302.2
20	ForQ+	FOR	Q+	2	387	3.02	0.2	5.23	6.17	0.87	23.8	43.2	33.9	1.13246	400
25	ForQ+	FOR	Q+	2	403	4.64	0.34	2.02	7.23	0.95	23.9	42.8	25.7	1.13433	248.95

27	ForQ+	FOR	Q+	2	307	3.4	0.24	3.91	5.31	0.67	24.6	44.9	28	0.40299	370.7
30	ForQ+	FOR	Q+	2	484	3.7	0.13	2.66	4.12	0.66	24.8	46.4	58.6	0.26306	374.3
1	ColQ-	COL	Q-	4	179	5.72	0.24	0.97	4.89	1.58	23	59.3	5.7	0.50933	220.8
4	ColQ-	COL	Q-	4	179	3.89	0.31	0.88	6.23	0.95	24.3	56.9	18.1	0.16604	231.05
5	ColQ-	COL	Q-	4	371	3.66	0.43	1.58	5.34	1.8	23.4	60.9	7.7	0.39925	167.55
10	ColQ-	COL	Q-	4	345	3.37	0.52	0.68	5.5	1.47	23.1	61.4	12.9	0.41045	191.55
12	ColQ-	COL	Q-	4	710	2.8	0.77	0.9	5.74	1.15	25.5	59.7	14.6	0.19216	145.95
23	ColQ-	COL	Q-	4	139	3.89	0.23	0.98	5.96	1.16	23.2	60.2	20	0.41978	227.3
28	ColQ-	COL	Q-	4	110	2.74	0.29	1.07	5.14	1.28	24.5	52.7	13.2	0.25933	172.7
2	ColQ+	COL	Q+	4	147	4.39	0.62	0.78	5.45	1.69	23.1	65.6	2.9	0.31903	187.35
3	ColQ+	COL	Q+	4	346	7.45	0.42	0.72	5.86	1.34	21.7	64.1	10.1	0.40299	239.55
7	ColQ+	COL	Q+	4	408	2.84	0.53	1.07	6.26	1.65	24	64.6	10.4	0.32276	189.8
8	ColQ+	COL	Q+	4	179	4.09	0.12	1.22	5.82	1.54	25.3	62.3	5.6	0.38246	160.5
11	ColQ+	COL	Q+	4	211	2.36	0.74	1.27	5.32	1.09	24.6	62.7	14.6	0.28358	164.45
19	ColQ+	COL	Q+	4	417	3.47	0.34	1.08	4.88	1.76	26.3	63.3	14.7	0.16418	157.15
26	ColQ+	COL	Q+	4	177	2.62	0.23	0.81	5.42	1.51	24.6	56.8	8.8	0.18097	165.3
9	ForQ-	FOR	Q-	4	227	4.18	0.36	0.48	3.55	1.04	20.6	49.5	3.3	0.26306	156.9
15	ForQ-	FOR	Q-	4	130	3.41	0.3	0.33	4.91	1.3	24.9	47.6	14.4	0.78731	101.85
18	ForQ-	FOR	Q-	4	95	4.5	0.16	1.63	4.98	0.85	22	42.5	11.1	0.20709	112.7
21	ForQ-	FOR	Q-	4	113	3.47	0.07	0.72	4.74	1.07	22.7	41.9	14.1	0.6903	106.55
22	ForQ-	FOR	Q-	4	91	3.15	0.1	0.66	4.96	1.02	22.8	40.7	14	0.1903	112
24	ForQ-	FOR	Q-	4	569.5	3.02	0.26	1.075	3.63	0.985	23.85	44.9	3.5	0.09328	164
29	ForQ-	FOR	Q-	4	356	4.9	0.23	1.76	3.87	0.77	22.5	44.1	20.1	0.24813	152.6
13	ForQ+	FOR	Q+	4	315	7.76	0.18	2.47	3.81	0.58	23.5	44.3	9	0.28358	85.05
14	ForQ+	FOR	Q+	4	73	5.9	0.09	1.23	4.66	0.67	20.8	42.3	6.6	0.41791	55.25
17	ForQ+	FOR	Q+	4	211	2.7	0.19	0.91	3.86	0.95	23.3	45.8	8	0.21828	170.9
20	ForQ+	FOR	Q+	4	692	2.6	0.27	1.57	4	0.83	24.6	45.6	31.1	0.09328	227.25
25	ForQ+	FOR	Q+	4	55	4.26	0.25	1.32	5.86	1.15	22.4	41.3	11.6	0.36567	136
27	ForQ+	FOR	Q+	4	.	.	.	.	.	.	.	.	.	.	.
30	ForQ+	FOR	Q+	4	325	5.77	0.17	0.72	3.64	0.93	21.8	44	30	0.1847	159.6
1	ColQ-	COL	Q-	7	236	6.18	0.26	0.4	5.07	2.1	22.6	56.2	12.6	0.34515	194.45
4	ColQ-	COL	Q-	7	105	5.65	0.18	0.44	5.73	1.05	23.3	51.2	4.9	0.46642	206.65
5	ColQ-	COL	Q-	7	145	4.58	0.11	0.55	5.37	2.07	21.4	49.9	14.7	0.42724	141.55
10	ColQ-	COL	Q-	7	133	4.69	0.26	0.47	4.88	1.52	22.3	55.5	10.8	0.28545	177.9
12	ColQ-	COL	Q-	7	233	4.15	0.24	0.47	5.26	1.07	23.2	49	8.1	0.20522	124.3
23	ColQ-	COL	Q-	7	93	4.73	0.21	0.48	5.44	1.15	23.1	57.2	8	0.45149	143.1
28	ColQ-	COL	Q-	7	118	5.06	0.2	0.55	4.93	1.47	23.2	50.2	10.2	0.33022	150.45
2	ColQ+	COL	Q+	7	117	6.89	0.23	1.62	5.32	2.26	22.4	59.9	7.7	0.28545	183.9
3	ColQ+	COL	Q+	7	162	6.62	0.24	0.32	5.17	1.56	20.6	57.3	5.2	0.47201	160
7	ColQ+	COL	Q+	7	134	5.9	0.1	0.51	6.08	1.71	23.5	58	6.1	0.375	169.2
8	ColQ+	COL	Q+	7	.	.	.	.	.	.	.	.	.	.	.
11	ColQ+	COL	Q+	7	141	4.31	0.34	1.17	4.99	1.48	23	53.8	7.5	0.32836	124.55
19	ColQ+	COL	Q+	7	153	4.04	0.26	0.8	5.08	1.71	23.5	52	10.9	0.52985	182.3
26	ColQ+	COL	Q+	7	234	4.38	0.16	0.53	4.61	1.43	23.6	52.8	11	0.125	154.9
9	ForQ-	FOR	Q-	7	316	5.15	0.18	0.5	4.52	1	21	53.2	1.3	0.18284	77.95
15	ForQ-	FOR	Q-	7	179	3.32	0.15	0.53	4.75	1.45	23.1	44.4	9.6	2.21455	55.6
18	ForQ-	FOR	Q-	7	511	6.34	0.07	0.4	3.47	0.5	22.7	40.2	9.1	0.09328	89.45
21	ForQ-	FOR	Q-	7	100	2.98	0.07	0.55	4.46	0.92	23.6	40	9.3	0.15299	75.15
22	ForQ-	FOR	Q-	7	87	5.28	0.08	0.04	5.01	1.07	21.5	38.4	7.2	0.24254	81.05
24	ForQ-	FOR	Q-	7	100	3.54	0.11	0.74	4.92	1.2	23.6	42.8	10	0.33209	80.45
29	ForQ-	FOR	Q-	7	95	4.58	0.06	1.35	3.86	1.06	20.7	38.7	10.4	0.36381	111.65
13	ForQ+	FOR	Q+	7	239	3.8	0.11	0.39	4.06	0.69	21.8	41.4	11.7	0.13993	46.4
14	ForQ+	FOR	Q+	7	153	5.2	0.11	0.28	5.05	0.82	21.8	42.3	8.7	0.35634	47.6
17	ForQ+	FOR	Q+	7	290	3.3	0.1	0.45	3.95	0.98	23	43.7	12	0.09328	95.5
20	ForQ+	FOR	Q+	7	90	3.36	0.13	0.46	5.28	0.89	22	38.4	20.5	0.28731	120.4

25	ForQ+	FOR	Q+	7	80	4.14	0.03	0.5	4.82	1.2	21.6	37.3	5.4	0.22575	69.05
27	ForQ+	FOR	Q+	7	159	3.65	0.07	0.63	5.25	1.08	21.5	38.4	11.2	0.20336	54.05
30	ForQ+	FOR	Q+	7 .	.	.	.	.	.	.	.	.	.	.	
