# Supplementary material for: Quercetin Feeding in Newborn Dairy Calves Cannot Compensate Colostrum Deprivation: Study on Metabolic, Antioxidative and Inflammatory Traits
Source: PLoS One. 2016 Jan 11;11(1):e0146932. doi: 10.1371/journal.pone.0146932 (PMC4709053; doi:10.1371/journal.pone.0146932)
Supplement: S4 Table — (PDF) [file pone.0146932.s004.pdf]

calf	group	feeding	quercetin	day of life	IgG1, g/L	IgG2, g/L	IgM, g/L	Fibrinogen, g/L	Haptoglobin, g/L	serum amyloid A, mg/L
1	ColQ-	COL	Q-	1	0	0	37.29	3.81	0	14.888
4	ColQ-	COL	Q-	1	0	0	30.82	0	0	13.438
5	ColQ-	COL	Q-	1	0	8.65	27.15	0	0	1.208
10	ColQ-	COL	Q-	1	0	11.15	25.41	2.17	0	26.579
12	ColQ-	COL	Q-	1	0	3.04	20.08	3.5	0	33.281
23	ColQ-	COL	Q-	1	0	9.81	48.12	2.86	0	14.13
28	ColQ-	COL	Q-	1	0	6.53	52.32	2.22	0	20.482
2	ColQ+	COL	Q+	1	0	4.53	13.46	2.71	0	1.499
3	ColQ+	COL	Q+	1	0	.	.	0.81	0	16.725
7	ColQ+	COL	Q+	1	0	8.1	72.09	0	0	0
8	ColQ+	COL	Q+	1	0	23.71	57.07	4.52	0	29.727
11	ColQ+	COL	Q+	1	0	0	20.25	3.75	0	9.764
19	ColQ+	COL	Q+	1	0	19.59	342.08	2.22	0	27.851
26	ColQ+	COL	Q+	1	0	5.35	20.82	1.67	0	13.159
9	ForQ-	FOR	Q-	1	.	.	129.64	5.24	0	107.84
15	ForQ-	FOR	Q-	1	.	.	27.75	3.49	0	18.943
18	ForQ-	FOR	Q-	1	0	5.32	10.54	1.95	0	36.547
21	ForQ-	FOR	Q-	1	0	4.19	44.36	4.35	0	22.804
22	ForQ-	FOR	Q-	1	0	5.01	18.69	1.46	0	17.747
24	ForQ-	FOR	Q-	1	0	5.41	39.22	2.86	0	14.659
29	ForQ-	FOR	Q-	1	0	5.07	42.59	2.6	0	23.306
13	ForQ+	FOR	Q+	1	0	4.37	16.99	2.86	0	26.197
14	ForQ+	FOR	Q+	1	0	2.97	26.66	3.25	0	40.492
17	ForQ+	FOR	Q+	1	0	91.95	48.33	2.1	0	32.56
20	ForQ+	FOR	Q+	1	0	5.32	22.17	1.96	0	23.143
25	ForQ+	FOR	Q+	1	0	3.2	15.97	2.04	0	17.394
27	ForQ+	FOR	Q+	1	0	7	15.31	2.34	0	21.011
30	ForQ+	FOR	Q+	1	0.01	7.81	105.56	1.84	0	18.453
1	ColQ-	COL	Q-	2	10.46	986.05	1386.49	4.6	0.07	139.162
4	ColQ-	COL	Q-	2	7.58	1021.1	726.9	3.42	0	126.353
5	ColQ-	COL	Q-	2	10.7	605.89	1151.22	2.86	0.05	200.51
10	ColQ-	COL	Q-	2	10.17	762.41	1234.9	4.88	0	168.81
12	ColQ-	COL	Q-	2	8.9	652.35	852.23	3.9	0	94.11
23	ColQ-	COL	Q-	2	10.46	573.12	1246.63	4.17	0	121.418
28	ColQ-	COL	Q-	2	9.6	665.97	752.27	3.04	0	90.493

2	ColQ+	COL	Q+	2	12.54	1381.67	1512.83	4.21	0	116.879
3	ColQ+	COL	Q+	2	13.43	1270.24	1661.41	2.86	0	119.036
7	ColQ+	COL	Q+	2	10.13	1316.29	1065.97	2.63	0	95.949
8	ColQ+	COL	Q+	2	9.47	1368.68	773.73	5.53	0.03	130.268
11	ColQ+	COL	Q+	2	10.13	906.28	1087.25	4.69	0	183.342
19	ColQ+	COL	Q+	2	6.4	589.25	865.14	3.02	0	128.851
26	ColQ+	COL	Q+	2	7.28	533.12	893.9	2.88	0	131.3
9	ForQ-	FOR	Q-	2	.	.	110.07	7.67	0	239.091
15	ForQ-	FOR	Q-	2	.	.	39.03	4.65	0	136.486
18	ForQ-	FOR	Q-	2	0	7.59	27.21	5.12	0	146.964
21	ForQ-	FOR	Q-	2	0	8.36	59.27	6.52	0	170.803
22	ForQ-	FOR	Q-	2	0	9.52	31.99	3.04	0	93.978
24	ForQ-	FOR	Q-	2	0	8.97	49.69	4.22	0	99.581
29	ForQ-	FOR	Q-	2	0	11.88	53.25	4.22	0.09	113.654
13	ForQ+	FOR	Q+	2	0	3.9	12.46	5.53	0.01	203.424
14	ForQ+	FOR	Q+	2	0	3.41	25.95	5.5	0.03	113.708
17	ForQ+	FOR	Q+	2	0	103.22	53.13	4.05	0.02	158.502
20	ForQ+	FOR	Q+	2	0	8.95	31.21	2.32	0	152.436
25	ForQ+	FOR	Q+	2	0	5.96	26.23	4.08	0	132.006
27	ForQ+	FOR	Q+	2	0	19.38	27	5.32	0	124.418
30	ForQ+	FOR	Q+	2	0.01	9.05	89.02	3.41	0	173.474
1	ColQ-	COL	Q-	4	8.51	638.67	922.58	5	0	91.405
4	ColQ-	COL	Q-	4	6.98	532.44	432.24	5.13	0	56.168
5	ColQ-	COL	Q-	4	11.86	620.97	881.6	7.32	0	222.568
10	ColQ-	COL	Q-	4	9.25	540.47	789.27	5.13	0	153.284
12	ColQ-	COL	Q-	4	8.6	424.71	549.25	5.23	0	98.394
23	ColQ-	COL	Q-	4	10.8	554.4	801.91	5.12	0	44.04
28	ColQ-	COL	Q-	4	9.25	674.06	440.74	3.69	0	83.346
2	ColQ+	COL	Q+	4	9.47	758.35	1108.26	7.43	0	125.193
3	ColQ+	COL	Q+	4	11.14	673	1032.83	4.42	0	51.286
7	ColQ+	COL	Q+	4	9.47	659.7	805.85	6.67	0	113.302
8	ColQ+	COL	Q+	4	9.8	916.82	478.51	5.58	0	61.533
11	ColQ+	COL	Q+	4	9.47	664.11	645.61	7.07	0	192.768
19	ColQ+	COL	Q+	4	6.98	511.52	765.53	4.65	0	112.308
26	ColQ+	COL	Q+	4	6.98	497.49	476.63	3.78	0	138.403
9	ForQ-	FOR	Q-	4	.	.	90.94	7.5	0.12	251.532

15	ForQ-	FOR	Q-	4	.	.	103.3	7.14	0	153.666
18	ForQ-	FOR	Q-	4	0	6.66	72.26	6.82	0.04	102.339
21	ForQ-	FOR	Q-	4	0	7.45	166.01	6.52	0	96.74
22	ForQ-	FOR	Q-	4	0	7.93	169.98	0.53	0	65.347
24	ForQ-	FOR	Q-	4	0	7.7	85.58	7.02	0	102.713
29	ForQ-	FOR	Q-	4	0.01	11.22	102.12	6.87	0.29	123.58
13	ForQ+	FOR	Q+	4	0	3.31	57.21	7.27	0.08	249.745
14	ForQ+	FOR	Q+	4	0	2.19	112.34	6.67	0.07	64.798
17	ForQ+	FOR	Q+	4	0	128.55	64.52	6.34	0.03	162.574
20	ForQ+	FOR	Q+	4	0	9.02	72.88	4.54	0	68.362
25	ForQ+	FOR	Q+	4	0	5.87	96.97	5.1	0	111.713
27	ForQ+	FOR	Q+	4	0	8.09	85.65	3.12	0	134.697
30	ForQ+	FOR	Q+	4	0.01	7.87	263.18	7.08	0.14	160.24
1	ColQ-	COL	Q-	7	7.89	630.33	555.9	3.9	0	106.728
4	ColQ-	COL	Q-	7	7.28	550.14	297.2	3.95	0	56.409
5	ColQ-	COL	Q-	7	9.8	841.64	426.18	0	0	104.311
10	ColQ-	COL	Q-	7	12.93	503.05	475.9	4.28	0	85.881
12	ColQ-	COL	Q-	7	8.8	534.17	424.38	3.02	0	105.054
23	ColQ-	COL	Q-	7	9.47	516.66	532.58	3.96	0	82.552
28	ColQ-	COL	Q-	7	7.84	491.83	388.82	3.57	0	69.185
2	ColQ+	COL	Q+	7	9.47	894.82	759.6	4.62	0	130.413
3	ColQ+	COL	Q+	7	13.98	694.06	955.99	3.25	0	17.208
7	ColQ+	COL	Q+	7	9.15	726.69	586.46	4.05	0	40.651
8	ColQ+	COL	Q+	7	9.47	849.34	335.79	4.5	0	166.134
11	ColQ+	COL	Q+	7	6.69	615.4	419.04	7.5	0	98.994
19	ColQ+	COL	Q+	7	6.4	501.25	518.34	2.13	0	115.404
26	ColQ+	COL	Q+	7	6.98	524.29	395.64	3.62	0	135.623
9	ForQ-	FOR	Q-	7	.	.	595.76	7.32	0.51	234.857
15	ForQ-	FOR	Q-	7	.	.	129.49	4.77	0	95.298
18	ForQ-	FOR	Q-	7	0	6.7	150.86	4.39	0.15	74.682
21	ForQ-	FOR	Q-	7	0	9.24	208.17	4.13	0	84.651
22	ForQ-	FOR	Q-	7	0	7.31	137.5	3.62	0	38.79
24	ForQ-	FOR	Q-	7	0	7.38	171.53	4.03	0	40.025
29	ForQ-	FOR	Q-	7	0.01	11.33	340.5	4.31	0.01	84.758
13	ForQ+	FOR	Q+	7	0	3.07	333.9	5.36	0.05	198.715
14	ForQ+	FOR	Q+	7	0	5.13	314.56	4.39	0	89.401

17	ForQ+	FOR	Q+	7	0	219.75	150.65	4.88	0	102.721
20	ForQ+	FOR	Q+	7	0	8.63	170.05	2.55	0	115.235
25	ForQ+	FOR	Q+	7	0	7.73	186.71	3.67	0	56.304
27	ForQ+	FOR	Q+	7	0	10.59	165.77	4.89	0	33.937
30	ForQ+	FOR	Q+	7	0.01	6.89	774.94	5.53	0.16	147.578
