# Supplementary material for: Quercetin Feeding in Newborn Dairy Calves Cannot Compensate Colostrum Deprivation: Study on Metabolic, Antioxidative and Inflammatory Traits
Source: PLoS One. 2016 Jan 11;11(1):e0146932. doi: 10.1371/journal.pone.0146932 (PMC4709053; doi:10.1371/journal.pone.0146932)
Supplement: S5 Table — (PDF) [file pone.0146932.s005.pdf]

calf	group	feeding	quercetin	time	FRAP, ASCE (µmol/L)	TEAC, TE (mmol/L)	TBARS, MDAE (µmol/L	Isoprostane, ng/L
1	ColQ-	COL	Q-	1	134.6154	1.6819	0.2333	872.8109
4	ColQ-	COL	Q-	1	165.3846	1.7668	0.4218	3323.5132
5	ColQ-	COL	Q-	1	113.0769	1.7610	0.2028	1845.8929
10	ColQ-	COL	Q-	1	191.5476	1.9957	1.6936	3432.0776
12	ColQ-	COL	Q-	1	155.9524	2.0034	1.0969	1497.9342
23	ColQ-	COL	Q-	1	153.7778	1.5671	0.3147	1316.6672
28	ColQ-	COL	Q-	1	164.6667	1.4825	0.4983	2079.7811
2	ColQ+	COL	Q+	1	106.9231	1.8702	0.2998	2065.6675
3	ColQ+	COL	Q+	1	133.8462	1.7879	0.3933	2070.5921
7	ColQ+	COL	Q+	1	151.1905	1.6585	0.1657	1115.4069
8	ColQ+	COL	Q+	1	221.9048	1.8550	0.4532	1855.9122
11	ColQ+	COL	Q+	1	126.4286	2.0747	0.8907	3667.3396
19	ColQ+	COL	Q+	1	144.4444	1.5086	0.6139	769.8432
26	ColQ+	COL	Q+	1	148.6667	1.5472	0.2622	818.9534
9	ForQ-	FOR	Q-	1	267.1429	2.1022	0.0711	2352.3347
15	ForQ-	FOR	Q-	1	143.3333	1.5558	0.6139	1097.7122
18	ForQ-	FOR	Q-	1	102.6667	1.4700	0.7206	917.4523
21	ForQ-	FOR	Q-	1	149.5556	1.4985	0.5625	2462.5512
22	ForQ-	FOR	Q-	1	136.8889	1.3872	0.1049	391.0270
24	ForQ-	FOR	Q-	1	121.7778	1.4995	0.0787	704.2284
29	ForQ-	FOR	Q-	1	132.8889	1.5868	0.1049	5267.3945
13	ForQ+	FOR	Q+	1	166.4444	1.5497	0.6406	573.8642
14	ForQ+	FOR	Q+	1	136.4444	1.4652	0.4270	812.8169
17	ForQ+	FOR	Q+	1	181.7778	1.5729	0.2669	1320.7710
20	ForQ+	FOR	Q+	1	168.5000	1.4656	0.4270	1118.5693
25	ForQ+	FOR	Q+	1	127.1111	1.4248	0.2360	1937.4495
27	ForQ+	FOR	Q+	1	125.1111	1.4689	0.2098	3083.5188
30	ForQ+	FOR	Q+	1	86.2222	1.5295	0.0787	2385.7651
1	ColQ-	COL	Q-	4	154.6154	1.9924	0.4200	681.7810
4	ColQ-	COL	Q-	4	108.7179	2.1655	0.3915	1110.7583
5	ColQ-	COL	Q-	4	102.0513	2.1962	0.4263	866.6392
10	ColQ-	COL	Q-	4	101.9048	2.2487	1.3848	2145.2079
12	ColQ-	COL	Q-	4	132.6190	2.2176	0.7569	1343.3708
23	ColQ-	COL	Q-	4	118.2222	1.8955	0.5769	637.0641
28	ColQ-	COL	Q-	4	126.2222	1.7902	0.4983	1350.5488
2	ColQ+	COL	Q+	4	137.3077	2.3243	0.6110	1351.5856
3	ColQ+	COL	Q+	4	278.4615	2.1981	0.4930	808.6144
7	ColQ+	COL	Q+	4	102.1429	2.0439	0.5953	607.5221
8	ColQ+	COL	Q+	4	133.8095	2.0946	0.3063	1079.3036
11	ColQ+	COL	Q+	4	89.2857	2.2734	1.3602	1860.1428
19	ColQ+	COL	Q+	4	135.5556	1.8518	0.7740	1287.5254
26	ColQ+	COL	Q+	4	126.2222	1.8160	0.2885	1532.5689

9	ForQ-	FOR	Q-	4	107.6190	2.2030	0.3549	1702.9267
15	ForQ-	FOR	Q-	4	96.0000	1.8628	0.5071	706.0256
18	ForQ-	FOR	Q-	4	124.2222	1.6815	0.3470	717.9126
21	ForQ-	FOR	Q-	4	114.0000	1.6724	0.3482	533.4366
22	ForQ-	FOR	Q-	4	115.3333	1.6858	0.1836	529.8769
24	ForQ-	FOR	Q-	4	106.0000	1.6264	0.2098	482.1558
29	ForQ-	FOR	Q-	4	.	1.7021	0.3147	2301.7404
13	ForQ+	FOR	Q+	4	144.4444	1.6053	0.2269	1248.0384
14	ForQ+	FOR	Q+	4	140.8889	1.6428	0.1868	1011.2009
17	ForQ+	FOR	Q+	4	127.5556	1.7106	0.1868	730.7388
20	ForQ+	FOR	Q+	4	204.0000	1.6147	0.3203	942.8628
25	ForQ+	FOR	Q+	4	97.3333	1.7235	0.2098	321.7425
27	ForQ+	FOR	Q+	4	.	.	.	.
30	ForQ+	FOR	Q+	4	133.5556	1.6842	0.1573	1802.3673
1	ColQ-	COL	Q-	7	131.5385	1.9982	0.6214	742.7139
4	ColQ-	COL	Q-	7	136.9231	2.1444	0.5979	1135.8663
5	ColQ-	COL	Q-	7	98.4615	2.1444	0.4627	703.2491
10	ColQ-	COL	Q-	7	123.8095	2.2211	0.8546	1904.1895
12	ColQ-	COL	Q-	7	124.5238	2.1732	0.8156	2309.5399
23	ColQ-	COL	Q-	7	102.8889	1.8389	0.3409	598.8129
28	ColQ-	COL	Q-	7	131.7778	1.8147	0.5769	1463.0938
2	ColQ+	COL	Q+	7	103.4615	2.2428	0.4065	1155.6262
3	ColQ+	COL	Q+	7	130.0000	2.1463	0.6163	703.7164
7	ColQ+	COL	Q+	7	162.8571	2.0908	0.3110	985.0263
8	ColQ+	COL	Q+	7				
11	ColQ+	COL	Q+	7	102.6190	2.1678	0.8112	1920.3906
19	ColQ+	COL	Q+	7	119.7778	1.8004	0.3470	565.4206
26	ColQ+	COL	Q+	7	141.5556	1.7395	0.2360	1562.8824
9	ForQ-	FOR	Q-	7	76.6667	2.1764	0.6622	2730.6530
15	ForQ-	FOR	Q-	7	99.1111	1.7905	0.4537	872.2011
18	ForQ-	FOR	Q-	7	94.2222	1.5563	0.3470	4620.7675
21	ForQ-	FOR	Q-	7	93.5556	1.5810	0.3482	556.8655
22	ForQ-	FOR	Q-	7	99.7778	1.5716	0.2622	607.7064
24	ForQ-	FOR	Q-	7	102.8889	1.7201	0.2098	508.0535
29	ForQ-	FOR	Q-	7	112.6667	1.6427	0.3409	2413.2883
13	ForQ+	FOR	Q+	7	.	.	0.1868	420.7918
14	ForQ+	FOR	Q+	7	91.5556	1.7406	0.1601	527.6681
17	ForQ+	FOR	Q+	7	98.8889	1.7077	0.3203	713.2756
20	ForQ+	FOR	Q+	7	.	1.5396	0.3737	900.2004
25	ForQ+	FOR	Q+	7	94.4444	1.6040	0.8392	1540.9193
27	ForQ+	FOR	Q+	7	80.8889	1.6122	0.3147	2460.9597
30	ForQ+	FOR	Q+	7	.	.	.	.
