# Supplementary material for: Quercetin Feeding in Newborn Dairy Calves Cannot Compensate Colostrum Deprivation: Study on Metabolic, Antioxidative and Inflammatory Traits
Source: PLoS One. 2016 Jan 11;11(1):e0146932. doi: 10.1371/journal.pone.0146932 (PMC4709053; doi:10.1371/journal.pone.0146932)
Supplement: S6 Table — (PDF) [file pone.0146932.s006.pdf]

				targets of interest										reference genes			
calf	group	feeding	quercetin	CAT	GPX	SOD	CRP	IL1A	SAA2	TNF	FGA	Hp	IL1B	LRP10	POL2A	beta_actin	HPCAL1
1	ColQ-	COL	Q-	1.17143	1.06366	1.15192	0.7976	1.23514	0.62615	0.39087	3.82877	14.275	3.3734	1.11062	1.32371	1.78423	2.98448
4	ColQ-	COL	Q-	1.09433	1.00378	0.90348	0.718	1.1661	0.53025	1.14482	1.44347	6.0121	4.12951	1.30542	1.56374	1.28497	1.50192
5	ColQ-	COL	Q-	1.26334	1.38293	1.19406	0.89045	1.3085	0.73128	0.53663	4.34262	22.7932	4.76277	1.19393	1.85821	1.18233	3.27089
10	ColQ-	COL	Q-	1.68193	1.07982	1.16457	0.87412	0.84428	1.06089	0.99429	1.47065	4.1538	1.53206	1.62734	1.50812	1.0688	1.56687
12	ColQ-	COL	Q-	0.98027	0.93044	0.8111	1.04122	1.27395	1.4042	1.35465	1.71678	21.3998	2.12321	2.44541	1.07265	1	1.62079
23	ColQ-	COL	Q-	1.13387	1.0099	0.93922	1.09176	1.18664	1.12075	0.94523	3.57125	19.7832	2.06005	1.20449	1.28786	1.69099	3.03024
28	ColQ-	COL	Q-	.	.	.	.	.	.	.	.	.	.	.	.	.	.
2	ColQ+	COL	Q+	0.73922	0.64333	1.02115	0.63041	1.29465	0.86595	1.69928	1.25948	19.6812	2.20152	1.80704	1.36213	1.06567	1.01059
3	ColQ+	COL	Q+	0.44354	1.25023	1.08499	1.02144	0.45686	0.8613	0.74644	3.56168	48.5291	2.98425	1.01548	1.62021	1.59429	1.49852
7	ColQ+	COL	Q+	0.94772	0.81753	0.92369	0.95628	0.99084	0.43953	0.74649	5.33002	24.5246	2.57569	1.60655	1.48464	1.09975	2.40735
11	ColQ+	COL	Q+	0.76525	0.92833	1.04868	1.09882	0.80116	1.29573	1.34743	2.70274	39.7927	3.44802	1.14434	1.45122	1.57952	1.99151
19	ColQ+	COL	Q+	0.54456	0.95776	1.12808	1.13751	0.56584	1.03359	1.47307	3.84848	36.3452	7.37029	1.04446	1.66645	1.50704	2.24306
26	ColQ+	COL	Q+	1.20786	1.0994	1.27642	0.89764	1.27159	0.80903	0.87578	2.99053	5.9909	3.52465	1	1.33272	1.9682	2.53918
9	ForQ-	FOR	Q-	0.88953	0.93322	1.30995	1.39457	0.93068	1.51382	0.76048	6.07127	97.2782	3.19267	1.03933	1.27779	1.97514	2.13348
15	ForQ-	FOR	Q-	1.27149	0.97648	1.3739	0.90225	1.33036	1.01482	0.58024	7.42837	57.9337	3.63976	1.27616	1.2705	1.61782	3.82103
18	ForQ-	FOR	Q-	1.53709	0.97762	1.26895	1.02068	0.347	1.67499	1.43352	1.43329	13.5158	2.81554	1.33933	1.07143	1.82794	1.50244
21	ForQ-	FOR	Q-	1.152	0.97053	0.83668	1.1456	1.2084	1.00005	0.79529	4.03869	22.7161	5.04311	1.30082	1.38654	1.45432	2.3124
22	ForQ-	FOR	Q-	0.7968	0.91963	0.80882	1.1498	1.00232	0.7198	1.22524	1.72817	9.4138	2.239	1.17379	1.41489	1.57941	1.52159
24	ForQ-	FOR	Q-	.	.	.	.	.	.	.	.	.	.	.	.	.	.
29	ForQ-	FOR	Q-	0.44006	1.0961	0.93117	1.05969	1.47852	1.28236	0.52395	1.70676	37.8081	1.72565	1.19509	1.27504	1.72141	1.14704
13	ForQ+	FOR	Q+	0.92814	1.11748	0.90468	0.94202	0.96915	1.82901	1.39742	4.00911	67.3544	5.47918	1.06309	1.67006	1.47743	2.43242
14	ForQ+	FOR	Q+	1.07636	1.2076	0.71457	0.92361	0.73109	1.15721	1.5481	1.34891	14.6176	2.11813	1.38901	1.11465	1.69421	1.40983
17	ForQ+	FOR	Q+	1.11213	0.8946	1.09038	0.93096	1.16911	1.03578	0.60566	3.4702	26.4486	2.42419	1.08941	1.4429	1.66873	2.19312
20	ForQ+	FOR	Q+	0.99311	0.98436	1.02362	0.9965	2.08857	1.37232	1.62735	1.80067	17.9123	3.68457	1.2915	1.27819	1.58899	1.68524
25	ForQ+	FOR	Q+	0.92992	0.88046	0.90038	1.3269	1.49731	0.6904	1.06856	1.46064	11.2364	1.4396	1.42924	1.27971	1.43415	1.75185
27	ForQ+	FOR	Q+	1.42541	1.21503	0.75697	1.21643	0.61317	0.65656	2.66255	1	1	1	1.43461	1.59914	1.14338	1.98326
